# Correlated insulator in the kagome flat band of a two-dimensional electrostatic crystal

**DOI:** 10.1038/s41567-026-03291-7

**Published:** 2026-05-11

**Authors:** Daisy Q. Wang, Zeb Krix, Olga A. Tkachenko, Vitaly A. Tkachenko, Chong Chen, Ian Farrer, David A. Ritchie, Oleg P. Sushkov, Alexander R. Hamilton, Oleh Klochan

**Affiliations:** 1 School of Physics, University of New South Wales, Sydney, New South Wales Australia; 2 Rzhanov Institute of Semiconductor Physics, Novosibirsk, Russia; 3 Novosibirsk State University, Novosibirsk, Russia; 4 Cavendish Laboratory, J. J. Thomson Avenue, Cambridge, UK; 5 School of Science, The University of New South Wales, Canberra, Australian Capital Territory Australia

**Keywords:** Electronic properties and materials, Electronic and spintronic devices

## Abstract

The electronic properties of solids are determined by their crystal structure and electron interactions, giving rise to phenomena such as superconductivity, strange metals and correlated insulators. Many of these effects remain poorly understood, motivating efforts to create artificial crystals that mimic real materials while allowing controlled tuning of key parameters. Cold atoms in optical lattices offer flexibility but cannot reproduce the long-range Coulomb interactions and hopping present in solids. Solid-state systems naturally support these features, although they suffer from tunability and flexibility issues. Here we demonstrate a highly tunable artificial crystal formed by superimposing a periodic electrostatic potential onto a two-dimensional electron gas in a shallow GaAs quantum well. This engineered lattice exhibits a band structure characteristic of the artificial triangular lattice, distinct from that of the underlying cubic crystal. Electronic transport measurements show a sign change in the Hall coefficient as the chemical potential sweeps through the artificial bands. The band structure can be continuously tuned to realize linear graphene-like and flat kagome-like bands within a single device. A strong insulating state emerges at half filling of the kagome flat band, consistent with interaction-driven behaviour. This tunability provides an opportunity to explore correlated quantum states in a controlled setting.

## Abstract (web summary)

A tunable artificial crystal in a shallow GaAs quantum well is shown to enable interaction-driven insulating behaviour. Electrostatic control tunes the band structure from graphene-like to kagome-like bands.

## Main

A key challenge in condensed-matter physics is understanding strongly interacting quantum systems where many-body correlated states such as superconductivity emerge. Artificial crystals, in which the key parameters can be controlled in situ, provide a powerful tool to simulate and study these complex systems. A variety of artificial crystal platforms have been developed, including electronic lattices in quantum wells, cold atoms in optical lattices, as well as photonic and plasmonic lattices^1^. While each of these approaches offers unique advantages, solid-state platforms stand out due to their natural incorporation of long-range Coulomb interactions, which are critical for emulating collective behaviour of real materials. However, creating solid-state artificial crystals is a non-trivial task. The major challenges lie in fabricating a highly uniform periodic potential *U*(*r*) with an amplitude much larger than the Fermi energy *E*_F_, while maintaining very low levels of disorder *Γ* ≪ *U*.

Most artificial solid-state crystals fall into two categories distinguished by the nature of the superlattice potential. In moiré superlattices, the periodic lattice potential is created by stacking atomically thin two-dimensional (2D) materials^2,3^. The interaction between different layers can lead to the formation of isolated flat bands where a diverse range of correlated electronic phases have been observed^4–8^.

An alternative approach involves artificially patterned superlattices imposed on conventional 2D systems, with the advantage that arbitrary lattice geometries can be created with excellent control. Unlike moiré superlattices, where flat bands emerge at discrete ‘magic’ twist angles due to non-Abelian gauge fields^9^, patterned electrostatic lattices rely on a scalar periodic potential whose strength can be continuously and adiabatically tuned. This tunability enables exploration of a wider parameter space and direct control of the band structure within a single device. While recent advances, such as the quantum twist microscope, have introduced in situ twist-angle control in moiré systems^10^, these approaches require complex scanning probe set-ups at ultralow temperatures. Moiré platforms are rapidly evolving and could in the future allow a wide range of lattice geometries and materials, including semimetals, atomically thin magnetic layers and 2D superconductors. Meanwhile, patterned lattices allow straightforward realization of a broad range of geometries with precise and reproducible control, providing an experimentally flexible and robust platform for studying correlated states.

Early studies of superlattices on doped semiconductor heterostructures revealed Weiss oscillations^11,12^ and signatures of Hofstadter physics^13–15^, but the weak artificial lattice potential (*U*(*r*) ≪ *E*_F_) and disorder prevented the formation of an artificial solid-state crystal. Recent optical studies of honeycomb lattices etched into GaAs quantum wells^16,17^ have revealed characteristics of the honeycomb bands^18^ and possible many-body effects^19,20^, but etched systems do not allow continuous tuning of the superlattice potential. In this work, we present a low-disorder 2D artificial crystal defined in semiconductor heterostructures by nanolithographical patterning of electrostatic gates. The flexibility of our design enables continuous tuning to form a graphene-like crystal, or a kagome-like crystal, within a single sample. The observation of an artificial electronic kagome lattice^21^, where destructive interference between electron wavefunctions induces an electronic flat band, will allow new studies of a wide range of exotic quantum phenomena^22^ and a rich variety of correlated effects^23^.

**Fig. 1:**
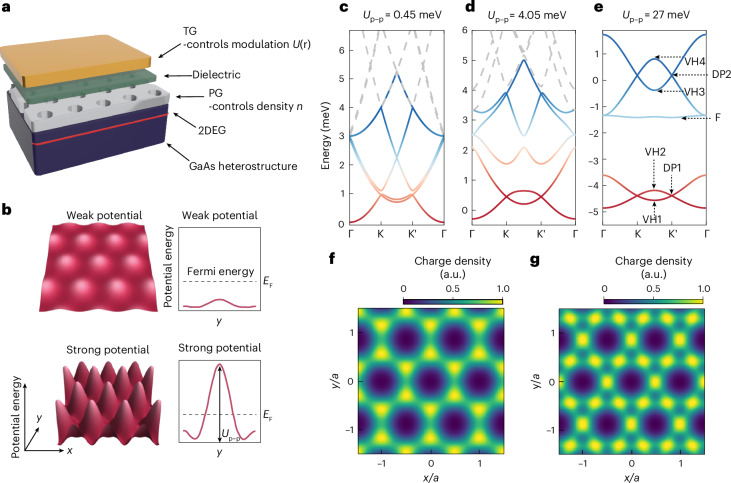
Tunable band structure of the electrically defined artificial crystal. **a**, Schematic of the device, showing the double-layer gate design. A surface metal PG (closest to the 2DEG) is patterned with a triangular array of holes and positively biased to induce electrons at the GaAs/Al_0.6_Ga_0.4_As heterointerface 25 nm below the gate. The lattice constant is *L* = 100 nm, and the hole diameter is 45 nm. The overall top gate (TG), separated by a thin AlO_*x*_ dielectric, controls the strength of the superlattice potential through the holes etched in the PG. A strong modulation potential *U*(*r*) is essential for creating an artificial crystal with well-defined band structure. **b**, The modulation potential in the weak (*U*_p−p_ < *E*_F_) and strong (*U*_p−p_ > *E*_F_) limits. **c**–**e**, Evolution of the calculated band structure for a 100-nm lattice spacing with different peak-to-peak modulation amplitudes *U*_p−p_. Colours in **c**–**e** are used for visualization purposes and do not represent a quantitative scale. For a weak modulation potential (*U* < *E*_F_, typically *E*_F_ ≈ 2.5 meV), the mini-bands have mostly parabolic energy dispersion, with small splittings near the artificial Brillouin zone boundary (**c**). As the strength of the modulation potential increases, the splittings at the zone boundaries become larger (initially only the lowest graphene-like bands are well defined); higher bands overlap and form a ‘spaghetti’ of intersecting levels (**d**). Only at very strong potential modulation do the mini-bands become distinct and non-overlapping. In addition to the two graphene-like bands at lower energies (red), there are three kagome-like bands at higher energies (blue), and special points in the band structure can be identified in experiments: the VH singularities at the band edges (VH1–VH4), the DPs at band crossings (DP1 and DP2) and the flat band (F) (**e**). **f**,**g**, The real-space charge distributions are calculated for the graphene-like bands at strong modulation strength (**f**) and for kagome-like bands at very strong modulation (**g**). For **f**, states with energy −0.3 meV < *ϵ* < *μ* = *E*_F_ = 1.5 meV in **d** contribute to the density. For **g**, states with energy −1.5 meV < *ϵ* < *μ* = *E*_F_ = −0.5 meV in **e** contribute to the density.

In Fig. 1a, we show a schematic of the dual gate device. Electron beam lithography is used to define a 100-nm period triangular lattice in a metal gate electrode only 25 nm above the GaAs/AlGaAs heterointerface. This electrode defines the lattice geometry and is used to vary the average carrier density (band filling). A second overall top gate is deposited on top of a thin dielectric above the patterned gate (PG), and controls the strength of the lattice modulation and, thus, the artificial band structure. The dual gate architecture and the small distance between the PG and the 2D electron gas greatly amplifies the superlattice potential, allowing us to reach the regime where *U*(*r*) ≫ *E*_F_. We eliminate random disorder from dopant atoms by using entirely undoped crystals to ensure *Γ* ≪ *E*_F_, *U* (see Supplementary Section I for a comparison of this approach with previous studies).

To calculate the artificial band structure, we model the lattice as the periodic potential shown in Fig. 1b) and described by equation (1) in Methods, and perform an exact numerical solution of the single-particle Schrödinger equation to obtain the artificial band structure (self-consistent numerical modelling has also been performed in refs. ^24,25^). The effective strength of the periodic potential is determined by the ratio of the peak-to-peak magnitude *U*_p−p_ to the Fermi energy *E*_F_, as sketched in Fig. 1b. When the superlattice potential is weak, the mini-bands are essentially parabolic (Fig. 1c), corresponding to nearly free electrons, a regime that has been studied extensively in experiments with GaAs superlattices^13–15,26^. To create an artificial crystal, the superlattice potential must be strong (*U*_p−p_ > *E*_F_). In this regime (Fig. 1d,e) the energy bands start to separate. Two graphene-like bands at low densities develop first when the modulation strength is strong enough (Fig. 1d). Going to even stronger modulation causes three kagome-like bands (blue) to develop at high energies (by ‘graphene-like’ and ‘kagome-like’ we mean that the energy dispersion matches that of the corresponding lattice with nearest-neighbour hopping). Charge density distributions calculated with the Fermi energy positioned within the graphene-like bands (Fig. 1f) or kagome-like bands (Fig. 1g) reveal that electrons form a ‘graphene-crystal’ or ‘kagome-crystal’ around the repulsive triangular antidot lattice potential *U*_p−p_.

The major difference between the artificial bands in Fig. 1e and those in natural materials is the smaller bandwidth *W* (meV instead of eV) due to the larger lattice constant, *L* = 100 nm. This necessitates very low levels of disorder but allows the Fermi level to be easily swept through the different bands by tuning the voltage on the PG. The artificial band structure and its topology can be detected through the dynamics of electrons in this artificial crystal. If the Fermi surface expands with increasing energy, the particles in the band are electron-like, whereas if the Fermi surface shrinks, then charge carriers are hole-like. Transitions between electron-like and hole-like dynamics can only occur at well-defined points in the band structure, namely, van Hove (VH) singularities or Dirac points (DP), as labelled in Fig. 1e. A change of carrier type will result in a change in the sign of the Hall coefficient (*R*_H_) with *R*_H_ < 0 indicating electron-like carriers and *R*_H_ > 0 indicating hole-like carriers. This provides a clear experimental signature in electrical transport measurements.

**Fig. 2:**
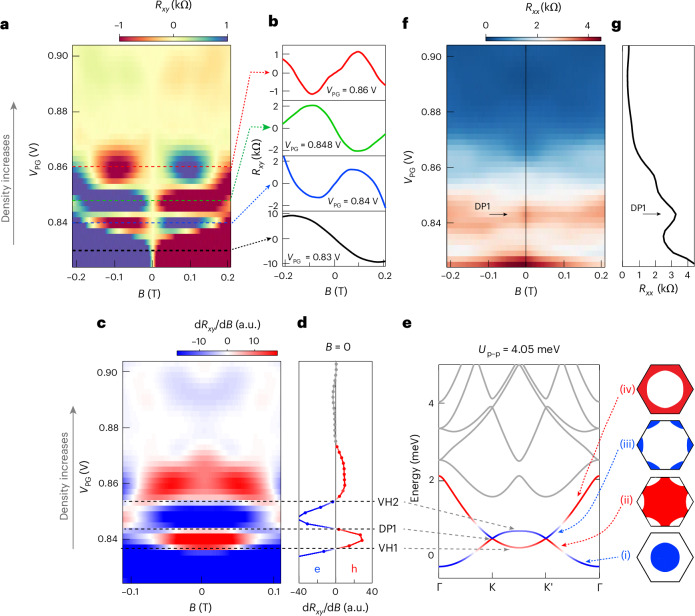
Measuring artificial band structure in graphene-like bands. **a**, Measured low-field Hall resistance *R*_*xy*_ of device D251 (Van der Pauw geometry with ~2,900 lattice sites) at *V*_TG_ = −0.5 V, *T* = 1.5 K. **b**, Line cuts of the Hall resistance at four different carrier densities indicated by the dashed lines in **a**. The Hall slope changes sign near *B* = 0: the black and green lines have a negative slope indicative of electron-like carriers, while the red and blue lines have a positive slope indicative of hole-like carriers. **c**, The transition points between electron-like and hole-like behaviours are highlighted, which plots the Hall coefficient *R*_H_ = d*R*_*xy*_/d*B*. Electron-like and hole-like behaviours are illustrated by the colours blue and red, respectively. Three transition points separate four regions of different carrier type, alternating between electron-like, hole-like, electron-like and hole-like as *V*_PG_ is increased. **d**, A line cut of the Hall coefficient at *B* = 0 is plotted with electron-like (negative) and hole-like (positive) region coloured with blue and red, respectively. **e**, This sequence of transitions is consistent with the calculated band structure, where the two graphene-like bands at low energy have three transition points: VH1, DP1 and VH2. The electron-like (blue) and hole-like (red) behaviour on either side of a transition is colour-coded, with (i)–(iv) showing the corresponding Fermi surfaces. **f**, Additional evidence for the formation of a DP comes from the longitudinal resistance *R*_*xx*_, plotted as a function of *V*_PG_ and magnetic field, *B*. **g**, A line cut of *R*_*xx*_ at *B* = 0 shows a clear resistance peak at the position of DP1 (indicated by the black arrow). Source data

Experimentally, we probe the formation of the artificial band structure using measurements of the low-field magnetoresistance at *T* = 1.5 K while continuously varying *V*_PG_ (Fig. 2). This positive bias on the PG is linearly proportional to the carrier density (band filling) as our device essentially functions as a field-effect transistor (Supplementary Section XI). As shown in Fig. 2a and highlighted by the line cuts in Fig. 2b, the slope of the Hall resistance near *B* = 0 undergoes a series of sign changes: from negative to positive, back to negative, and then to positive again as the carrier density increases. This behaviour is further illustrated by calculating the Hall coefficient *R*_H_ = d*R*_*xy*_/d*B* (Fig. 2c), where the line cut at *B* = 0 (Fig. 2d) shows switching between electron-like carriers (*R*_H_ < 0) and hole-like carriers (*R*_H_ > 0). In the artificial band structure, when the Hall coefficient changes from negative to positive, the carrier type transitions from electron-like to hole-like, indicating the presence of a VH singularity. Conversely, when the Hall coefficient changes from positive to negative, the carrier type transitions from hole-like to electron-like, signifying the presence of a DP. This sequence of varying carrier types—from electron-like to hole-like, back to electron-like, and then to hole-like again—aligns precisely with the calculated band structure at a modulation strength of *U*_p−p_ = 4.05 meV (Fig. 2e), where the three transition points at which *R*_H_ crosses zero are consistent with the positions of VH1, DP1 and VH2 in the calculated band diagram. From the sequence of Hall sign changes alone, a VH–gap–VH scenario could, in principle, also be considered. However, such a sequence does not occur in the calculated artificial band structure of a triangular lattice; instead, the observed features are uniquely consistent with a VH1–DP1–VH2 sequence. For high densities (above the last hole-like region, *V*_PG_ > 0.87 V), *R*_H_ is strongly suppressed when the mini-bands merge together. In this case, no clear signature of discrete bands is expected in the Hall slope (Supplementary Section IX). We use the point at which *R*_H_ is suppressed to estimate the value of *U*_p−p_, and because this is taken from experiments, it includes the effects of screening (Supplementary Section VIII).

Additional evidence for the formation of graphene-like bands comes from the evolution of the longitudinal resistance *R*_*xx*_ as a function of carrier density, controlled by *V*_PG_ (Fig. 2f,g). There is a clear resistance peak at *V*_PG_ = 0.843 V centred at the position of DP1, which is expected for a graphene-like system. We estimate the mobility of the charge carriers near the artificial Dirac cone using the resistance and effective density at VH1 or VH2 (0.6 × 10^10^ cm^−2^) to be *μ*_D_ ≈ 100,000 cm^2^ V^−1^ s^−1^, which is approximately ten times higher than that of the host 2D electron gas (2DEG) at the same carrier density. This high mobility is consistent with the linear dispersion of the graphene-like bands. We use the width of the Dirac peak to estimate the disorder, *Γ* ≲ 0.1 meV, which is 40 times smaller than the superlattice potential *U*_p−p_. Unambiguously distinguishing between massless Dirac fermions, massive Dirac fermions or a small gapped band edge is experimentally challenging and, in a strict sense, impossible, especially in artificial systems where disorder broadening is present. Nonetheless, the combination of high mobility, a clear resistance peak and the systematic evolution of transport features with superlattice modulation provides strong evidence for the formation of graphene-like bands in our artificial lattice.

**Fig. 3:**
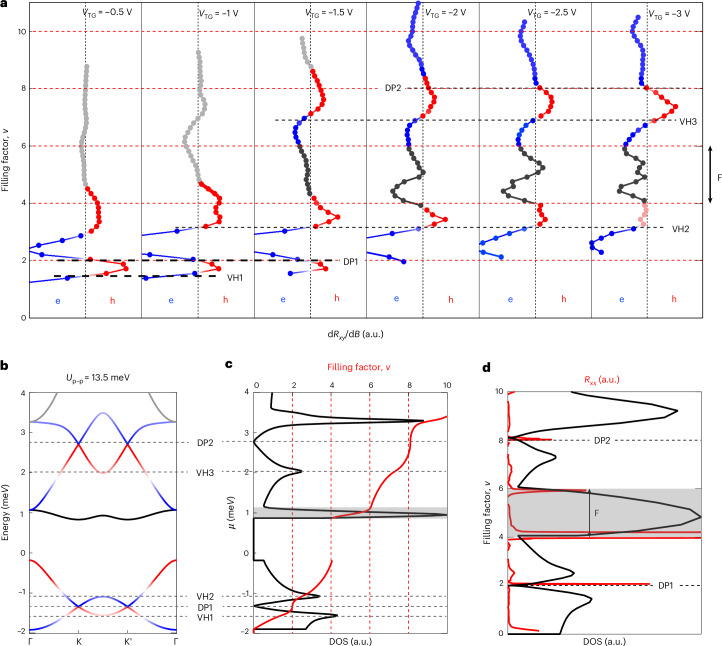
Tuning of the band structure and kagome-like bands. **a**, Measured low-field Hall coefficient of device D251 at *B* = 0 for different modulation strengths, plotted as a function of the filling factor (number of electrons per unit cell). From left to right: the superlattice modulation potential is increased as *V*_TG_ becomes more negative. Electron-like (negative) and hole-like (positive) Hall coefficient *R*_H_ = d*R*_*xy*_/d*B* are coloured blue and red, respectively. Dashed black lines indicate the positions of the VH singularities (VH1, VH2 and VH3), where the Hall coefficient crosses zero from negative to positive, as well as the DPs (DP1 and DP2, corresponding to one and four fully filled bands), where the Hall coefficient crosses zero from positive to negative. The section corresponding to populating the kagome flat band F (4 < *ν* < 6) is coloured black. In this section, the Hall coefficient is theoretically not well defined due to the narrow bandwidth. Data points are shown in a lighter shade of red for the region above VH2 at *V*_TG_ = −3V, where *R*_H_ remains slightly negative at *B* = 0 but becomes positive in a small magnetic field (Supplementary Section III). **b**, The calculated band structure for *U*_p−p_ = 13.5 meV shows both graphene-like and kagome-like bands (see Supplementary Section IX for details of the estimation of the superlattice potential strength). Electron-like and hole-like sections of the band structure are colour-coded with transition points labelled. **c**, Chemical potential corresponding to **b** as a function of DOS on the bottom axis (black) and filling factor *ν* on the top axis (red). Because the DOS is zero in the bandgap region from 0 to 1 meV, electron filling jumps directly into the band above. **d**, KWANT-simulated resistance *R*_*xx*_ (red) and corresponding DOS (black) as a function of the filling factor. In these single-particle calculations, the resistance peaks occur at DPs and band edges where the DOS is minimal. Source data

Having shown that we can use the electrostatic gate to transform 2D electrons in the GaAs quantum well into a graphene-like crystal, and established that the sign changes of the Hall coefficient at the DPs and VH singularities provide reliable anchor points for mapping the entire artificial band structure, we now exploit the ability to tune the strength of the superlattice potential to create a kagome lattice. Figure 3a shows the evolution of the Hall coefficient at *B* = 0 as the modulation strength is increased by applying a more negative voltage to *V*_TG_ (more details on band evolution can be found in Supplementary Section III). Here, we plot *R*_H_ as a function of the filling factor *ν*, the number of electrons per lattice unit cell (which is directly proportional to *V*_PG_). Because of spin, a full band has a capacity of two electrons per unit cell. The filling factor is calibrated from the spacing of the Hall sign changes, as detailed in Supplementary Section III. This filling factor assignment was verified through measurements of multiple devices on different wafers. To track the evolution of the band structure with *V*_TG_, we follow the experimentally determined transition points that separate the electron-like and hole-like regions, as marked in Fig. 3a. We found that as *V*_TG_ is made more negative, the increased modulation causes new sign changes of *R*_H_ to emerge at higher band fillings, while the previously identified sign changes associated with the VH1, VH2 and DP1 points in the lower graphene-like bands become suppressed. The order and spacing of these new *R*_H_ transitions are fully consistent with the expected formation of kagome-like bands as calculated in Fig. 3b, revealing the third VH singularities VH3, as well as DP2.

In addition to the new VH and DPs, the formation of kagome-like bands at the stronger modulation offers two more interesting features: one is the opening of a band gap between the kagome-like bands at higher energy and the Dirac-like bands at lower energy; the other is the formation of a kagome flat band with a narrow bandwidth (~0.2 meV), where strong correlation effects are expected. The Coulomb interaction energy scale, *e*^2^/*ϵL*, is about 1 meV. Because the bands are not completely flat, we can use the standard Wigner–Seitz radius formula to estimate 30 < *r*_s_ < 100 at quarter filling of the flat band.

This means that the strong correlation regime can be readily achieved in the kagome flat band when the potential modulation is strong. To fully understand the magneto-transport properties of the device in this strong modulation regime, we plot the density of states (DOS = d*N*/d*ϵ*) corresponding to Fig. 3b versus chemical potential in Fig. 3c and versus the filling factor in Fig. 3d. In the absence of disorder, there are no states within the band gap, so the chemical potential jumps over the band gap as shown by the red line in Fig. 3c. This is why the band gap collapses to a single point at *ν* = 4 in Fig. 3d. In reality, due to the existence of disorder and impurities, there are some states within the band gap. In our device, based on the density calibration, we estimate the disorder-related capacity of the band gap is less than 5% of the capacity of a band (Supplementary Section XI). Above the band gap, the DOS shows a strong spike in the kagome flat band(Fig. 3c), which could host possible correlated states. (There is another spike in the DOS at *μ* ≈ 3.3 meV; however, because both dispersing and non-dispersing electrons exist in this region, no correlated states are expected.) Despite the narrow bandwidth in energy (~0.2 meV), the flat band holds the same electron density as any other band, and has a filling factor of *ν* = 2. This is illustrated in Fig. 3d, which directly matches the transport measurement when the band filling is varied by *V*_PG_.

**Fig. 4:**
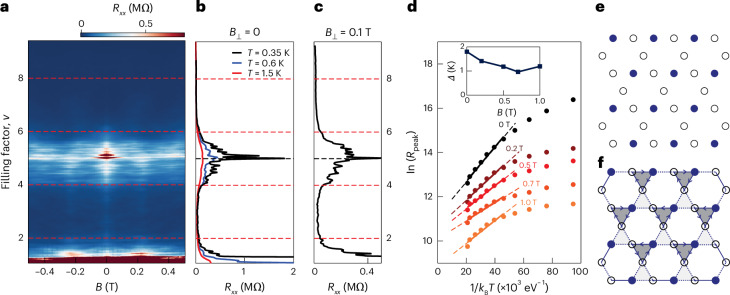
Insulating state in the flat band and loop-current Wigner insulator. **a**, Longitudinal resistance *R*_*xx*_ of device D252 (Hallbar geometry with ~600 lattice sites) with *V*_TG_ = −2 V at *T* = 350 mK. Dashed red lines indicate the band filling. **b**,**c**, Black traces show line cuts of *R*_*xx*_ at *B*_⊥_ = 0 (**b**) and *B*_⊥_ = 0.1 T (**c**); dashed black lines indicate half filling of the flat band. Blue and red traces in **b** show *R*_*xx*_ at elevated temperatures at *B*_⊥_ = 0. The sharp resistance peak at half filling of the flat band is markedly reduced at *T* = 0.6 K and completely absent by *T* = 1.5 K, leaving only a broad background feature. **d**, Arrhenius plot (circles) of the resistance of the half-filling insulating state at different *B*_⊥_. The solid lines (with dashed extrapolation) show the fitting with $\exp [\,-\varDelta /(2{k}_{{\rm{B}}}T)]$. Inset: the extracted thermal activation gap *Δ* as a function of *B*_⊥_. **e**,**f**, Schematics of the generalized Wigner insulator (**e**) and the loop-current Wigner insulator (**f**) state at half filling of the flat band (1/3 filling of the kagome lattice). Solid and dashed lines in **f** illustrate two possible configurations of the triangular loop currents. Arrows on the solid triangles indicate the direction of the loop currents, which is disordered at the measurement temperature in the absence of *B*_⊥_. Source data

In Fig. 4a, we show the longitudinal resistance *R*_*xx*_ measured on device D252 (Supplementary Section IV), where a Hall bar geometry is used to obtain more accurate measurements of *R*_*xx*_. At very low densities (low band filling factor *ν*), the sample is insulating due to disorder and Anderson localization. Increasing the carrier density causes a rapid reduction in *R*_*xx*_, as increasing the carrier density screens the static disorder. There is a substantial rise in *R*_*xx*_ when the kagome flat band starts to fill (4 < *ν* < 6), with a very sharp resistance spike at half filling of the flat band (*ν* = 5). This huge resistance spike cannot be explained by trivial disorder, as disorder would be most prominent at the band edges (*ν* = 4 and *ν* = 6). To confirm this, we perform calculations of the resistance expected from a single-particle picture using the open-source code KWANT. The simulation results, which include a weak random disorder, are shown in red in Fig. 3d and do not show a resistance spike at *ν* = 5. By contrast, the experimentally observed resistance peak occurs at half filling of the flat band, which points towards a correlated insulating state. This sharp resistance peak at *ν* = 5 is also highly reproducible across multiple devices (Supplementary Section VII), further supporting its intrinsic, correlated origin rather than disorder-driven mechanisms.

The resistance of the insulating state at *ν* = 5 is extremely sensitive to an out-of-plane magnetic field: it is strongly suppressed by a field of only *B*_⊥_ = 100 mT, as shown in Fig. 4c. Interestingly, even though the resistance peak is suppressed by a small *B*_⊥_, thermal activation measurements show that the the size of the energy gap is almost unaffected by the field, staying around 1 K up to *B*_⊥_ = 1T.

This insulating state at half filling of the kagome flat band corresponds to 1/3 of the sites of the kagome lattice being occupied, which cannot be described by a Mott insulator with only onsite repulsion. Insulating states at fractional fillings of triangular lattices in moiré systems have previously been associated with commensurate Wigner insulator states^8,27^. However, such commensurate Wigner insulators on a kagome lattice, as illustrated in Fig. 4e, cannot explain the observed strong dependence of *R*_*xx*_ on *B*_⊥_. A unique feature of the kagome lattice is that, in the commensurate Wigner insulator configuration, each electron is surrounded by empty sites with only one site occupied per kagome triangle (Fig. 4e). This special configuration at 1/3 filling of the kagome lattice facilitates electron delocalization across three neighbouring sites within a kagome triangle. Theoretically, this process is energetically favourable because it does not affect the Coulomb energy, but reduces the zero-point kinetic energy. This electron delocalization inevitably leads to a circulating current around the kagome triangle, conceptually resembling the loop-current model proposed for cuprates^28^.

The insulating state can be visualized as a series of in-plane loop currents on a triangular lattice as illustrated in Fig. 4f. Because each loop current carries a magnetic moment, the insulating state can also be interpreted as a set of orbital magnetic moments on a triangular lattice perpendicular to the plane (Ising type). The magnetic moment per loop is rather large, estimated to be ${\mu }_{\pm }=\pm \frac{et{L}^{2}}{16}\approx \pm 10{\mu }_{{\rm{B}}}$ where *L* = 100 nm is the lattice constant and *t* ≈ 0.6 meV is the nearest-neighbour hopping for the tight-binding kagome model. The value of *t* is estimated based on the band structure shown in Fig. 3b, with details provided in Supplementary Section XII. In a perfect tight-binding kagome model with long-range repulsion but no long-range hopping, the Ising orbital magnetic moments remain disordered down to *T* = 0. However, in reality, a small next neighbour hopping term ${t}^{{\prime} }\approx 0.07\,\mathrm{meV}$ induces a very weak antiferromagnetic interaction between orbital magnetic moments ${J}_{AF}\propto {t}^{{\prime} 2}\approx 10-20\,\mathrm{mK}$ (Supplementary Section XII). As a result, the system remains orbitally paramagnetic at experimentally accessible temperatures (we estimate our electron temperature is ~100 mK).

This ‘loop-current Wigner insulator’ model also explains the strong suppression of the resistance despite the activation gap being largely unaffected by the magnetic field. The large magnetic moment of the loop currents makes them easily orderable, even under the influence of a tiny magnetic field *B*_⊥_. Conductivity of the correlated state is provided only by electrons thermally excited over the correlation induced energy gap *Δ*. At *B*_⊥_ = 0, these electrons scatter due to exchange interaction from the thermal fluctuations of the disordered magnetic moments. Applying *B*_⊥_ orders the loop currents, reducing scattering and thereby increasing conductivity, but does not change the gap. This mechanism agrees well with the experimental observation that resistance decreases with the application of a small *B*_⊥_, while the size of the energy gap *Δ* remains largely unaffected.

In addition to the central peak at *ν* = 5 in Fig. 3a–c, there are small peaks within the flat band, which form a broad background. However, these are much smaller than the *ν* = 5 peak, and are not fully reproducible from device to device (Supplementary Section VII). We believe that the small peaks are related to different charge orderings in the kagome flat band: unlike Mott insulators, the correlated insulator we observe arises from Wigner crystallization driven by long-range Coulomb interactions. These long-range interactions enable a rich variety of crystallization patterns at different fractional fillings of the kagome lattice in the range *ν* = 4–6 (ref. ^29^), which leads to multiple overlapping resistance spikes. Because the 1/3 filling of the kagome lattice is the most stable charge configuration, the peak at *ν* = 5 is the most prominent, while smaller resistance peaks are more susceptible to disorder that pins specific charge configurations. This explains why the *ν* = 5 peak remains consistent across multiple devices, while smaller peaks vary between devices. This characteristic pattern of small peaks around the central peak fundamentally differs from the behaviour of a Mott insulator, which does not rely on long-range Coulomb interactions (in a Mott insulator, one would not expect to see a series of smaller peaks surrounding the central resistance peak). This difference explains why our experimental observations contrast with many graphene-based systems.

Interestingly, our theoretical estimates also predict that the electron spins align ferromagnetically with an effective Heisenberg ferromagnetic interaction *J*_F_ ≈ 1 K (Supplementary Section XII). This behaviour contrasts with the antiferromagnetic spin alignment typically observed in Mott insulators, where each lattice site is occupied. In our case, the correlated insulator emerges at a fractional filling of the kagome lattice, involving more than one orbital state. Consequently, spin ferromagnetism arises through the Goodenough–Kanamori–Anderson mechanism. These intriguing properties of the observed correlated state, hosted by the electronic kagome lattice, are quite different to states observed in moiré systems and open exciting opportunities for future experimental exploration.

In summary, we demonstrated highly tunable artificial crystals in solid-state systems that enable studies of physical phenomena driven by long-range hopping and strong Coulomb interactions. Using this method, we observed a strong insulating state in a kagome flat band, consistent with the model of a loop-current Wigner insulator. The discovery of this unique correlated state is particularly important, as a true kagome lattice is rarely realized in other artificial solid-state systems, and correlation effects in such systems have yet to be observed in transport experiments.

We emphasize that our approach not only allows lattices of any geometry to be created, but is also material agnostic, making it applicable to a variety of 2D systems, including atomically thin materials^29–32^. Furthermore, the technique can be extended to generate topological systems by introducing spin–orbit interactions through the use of valence band holes instead of conduction band electrons^25^, or extended to study exotic phases in honeycomb and kagome systems^23,33,34^ including ferrielectric and topological ferromagnetic states in the high magnetic field regime^35,36^. Overall, the ability to create arbitrary crystal geometries, with unprecedented control over topology, doping, spin–orbit interaction and superlattice potential, opens up the possibility of fabricating and studying an extensive variety of synthetic quantum matter.

## Methods

### Theoretical methods

To model the artificial crystal, we need to know the shape of the potential experienced by electrons in the 2DEG. A full three-dimensional (3D) numerical model of the device in Fig. 1a, including Hartree screening, has been used in ref. ^24^ to calculate the artificial band structure. To simplify the analysis, we construct a model Hamiltonian that depends on only a single parameter *W*, which represents the amplitude of the applied potential, yet captures the essential physics and produces a band structure in very good agreement with the full 3D numerical solution:

1 $$\begin{array}{rcl}H & = & \frac{{p}^{2}}{2{m}^{* }}+U({\bf{r}})\\ U({\bf{r}}) & = & 2W\left[\cos ({{\bf{G}}}_{1}\cdot {\bf{r}})+\cos ({{\bf{G}}}_{2}\cdot {\bf{r}})+\cos ({{\bf{G}}}_{3}\cdot {\bf{r}})\right]\end{array},$$

where **G**_1,2_ are the basic reciprocal vectors of the triangular lattice and **G**_3_ = **G**_2_ − **G**_1_ with $| {{\bf{G}}}_{i}| =4{\rm{\pi }}/\sqrt{3}a$. Note that the minimum of the potential is *U* = −3*W* and the maximum is *U* = 6*W*, such that the peak-to-peak potential amplitude *U*_p−p_ is

2 $$U_{{\mathrm{p}}-{\mathrm{p}}}=9W.$$

The lattice constant *a* is 100 nm in all calculations. Here, higher harmonics of the potential have been neglected. The validity of this Hamiltonian has been checked against fully numerical 3D finite element Poisson calculations in ref. ^24^. It is possible, however, to understand the form of equation (1) in terms of the following considerations. (1) At the level of the PG, the potential has the shape of a triangular array of (circular) hat functions. (2) According to the Poisson equation, electrons in the plane of the 2DEG experience this potential with Fourier components modified from *U*_*k*_ → e^−*kz*^*U*_*k*_, where *z* is the distance to the gate. (3) The fundamental harmonics have the form $\cos ({{\bf{G}}}_{{\bf{i}}}\cdot {\bf{r}})$, with $| {\bf{G}}| =4{\rm{\pi }}/\sqrt{3}a$, and higher harmonics are suppressed relative to these by a factor ${{\rm{e}}}^{-4{\rm{\pi }}/\sqrt{3}a}(\approx 0.1)$. (4) For *W* = 0, electrons in the 2DEG are described by a quadratic dispersion, *p*^2^/2m^*^, where m^*^ = 0.0667*m*_e_ is the effective mass of electrons in GaAs.

The band structures were computed by exact numerical diagonalization of the Hamiltonian in equation (1). To do this, we write equation (1) in the basis of plane wave states, $\left|{\bf{k}}\right\rangle ={{\rm{e}}}^{i{\bf{k}}\cdot {\bf{r}}}$. Because *U*(**r**) only mixes states that differ in momentum by ±**G**_*i*_ or ±**G**_2_, this basis can be restricted to states, $\left|{\bf{k}}+{{\bf{g}}}_{i}\right\rangle$, where **k** is a momentum within the first Brillouin zone and **g**_*i*_ is an arbitrary reciprocal lattice vector. We thus diagonalize the following matrix:

$$\langle {\bf{k}}+{{\bf{g}}}_{i}| H| {\bf{k}}+{{\bf{g}}}_{j}\rangle =\frac{{({\bf{k}}+{{\bf{g}}}_{i})}^{2}}{2m}{\delta }_{ij}+W\mathop{\sum }\limits_{n=1}^{3}\delta ({{\bf{g}}}_{i}-{{\bf{g}}}_{j}-{{\bf{G}}}_{n}),$$

where *δ*_*ij*_ is the Kronecker symbol. This matrix must be truncated such that the ∣**g**_*i*_∣ are smaller than some upper limit; the limit is chosen such that eigenvalues and eigenvectors are independent of the limit (for example, the limit **g** = *n***G**_1_ + *m***G**_2_ with ∣*n*∣, ∣*m*∣ ≤ 10 is more than large enough for the strongest potentials that we consider). Numerical diagonalization gives a set of energy levels *ε*_*n*_(**k**) (plotted in Fig. 1) and a set of corresponding Bloch functions *ψ*_*n*,**k**_(**r**). The charge densities shown in Figs. 2e and 3f are equal to ${\sum }_{{\mu }_{0} < {\varepsilon }_{n}({\bf{k}}) < \mu }| {\psi }_{n,{\bf{k}}}({\bf{r}}){| }^{2}$. For Fig. 2e, we sum over states from the bottom of band 1 to the mid-point of band 2 (*μ* = 1.5 meV). For Fig. 3f, we sum over states from the bottom of band 3 to the top of band 4, −0.5 meV < *ϵ* < 2.7 meV. Of course, in the latter case, bands 1 and 2 also contribute to the total charge density; however, these states are not physically relevant when *μ* is within the kagome-like bands, and we exclude them for the sake of clarity.

### Experimental methods

The devices presented in the main text are fabricated on an ultrashallow high-quality undoped GaAs/Al_0.6_Ga_0.4_As heterostructure comprising a 3-nm GaAs cap, a 22-nm AlGaAs layer and a thick GaAs buffer layer, grown by molecular beam epitaxy. N-type ohmic contacts (AuGe) to the heterostructure were thermally evaporated into etched pits and then annealed. The triangular lattice is patterned into the surface metal gate by electron-beam lithography and reactive ion etching with SF_6_. A 15-nm-thick AlO_*x*_ dielectric is deposited with atomic layer deposition to isolate the metal gates from each other and from the ohmic contacts^37^. The mean free path of electrons in the unpatterned wafer is 3 μm at *n* = 2 × 10^11^ cm^−2^, much larger than the lattice constant. Transport measurements were performed with standard lock-in techniques between 17 Hz and 33 Hz. All devices are cooled with all gates grounded. Because of the undoped heterostructure used, these devices are very stable between different cooldowns. Measurements are done on two types of device design: a Van der Pauw geometry with a patterned area of 5 × 5 μm and a Hallbar geometry with a 2-μm-wide channel.

## Online content

Any methods, additional references, Nature Portfolio reporting summaries, source data, extended data, supplementary information, acknowledgements, peer review information; details of author contributions and competing interests; and statements of data and code availability are available at 10.1038/s41567-026-03291-7.

## Supplementary information

Supplementary Information Supplementary Figs. 1–20, Tables 1 and 2 and discussion.

## Source data

Source Data Fig. 2 Source data for Fig. 2b,d,g.

Source Data Fig. 3 Source data for Fig. 3a–d.

Source Data Fig. 4 Source data for Fig. 4b–d.

## Data Availability

Other relevant data are available from the corresponding authors upon reasonable request. Source data are provided with this paper.
