# Supplementary material for: Correlated insulator in the kagome flat band of a two-dimensional electrostatic crystal
Source: Nat Phys. 2026 May 11;22(7):1079–86. doi: 10.1038/s41567-026-03291-7 (PMC13423788; doi:10.1038/s41567-026-03291-7)
Supplement: Supplementary file 1 — Supplementary Figs. 1–20, Tables 1 and 2 and discussion. [file 41567_2026_3291_MOESM1_ESM.pdf]

nature physics

Supplementary information https://doi.org/10.1038/s41567-026-03291-7

Correlated insulator in the kagome flat band of a two-dimensional electrostatic crystal

In the format provided by the authors and unedited

CONTENTS

I. Comparison of our device with previous studies 2

II. Measurements in the weak modulation regime 2

III. Bandstructure evolution and Calibration of the filling factor 4

IV. Reproducibility across multiple devices 7

V. Behaviour of the band gap 10

VI. Measurements of different configurations and temperatures on device D252 11

VII. Reproducibility of the correlated state and the role of disorder 14

VIII. Coulomb screening, effect on modulation strength 16

IX. Estimation of modulation strength 17

X. Estimation of disorder and mobility 19

XI. Estimation of quantum capacitance 21

XII. Loop Current Wigner Insulator 23

References 27

I. COMPARISON OF OUR DEVICE WITH PREVIOUS STUDIES

Creating an artificial crystal and observing mini-bands requires a highly uniform periodic potential with an amplitude exceeding the Fermi energy while minimizing disorder broadening of the energy levels. To highlight the advantages of our device architecture we compare the carrier mobility after patterning versus the artificial lattice constant in systems where the modulation potential is strong (Fig. S1(a)), including early studies on etched GaAs and graphene systems. While etching can create a strong modulation potential, it also introduces disorder, leading to unwanted scattering that limits the carrier mobility and prevents the observation of artificial bandstructure. In contrast, our patterned device maintains high carrier mobility, measuring $\sim 500,000$ cm^2/Vs at a high density 2×10^{11} cm^{-2}, where superlattice effects are negligible. The same measurement gives $\sim 300,000$ cm^2/Vs at 1.5×10^{11} cm^{-2}. For comparison, devices with an unpatterned 2D top gate exhibit similar mobility at these densities, confirming that our fabrication process introduces minimal damage to the heterostructure.

To highlight the tunability of our device architecture, we compare our system to others where artificial bands have been observed (Fig. S1(b)). We plot the number of accessible bands against the normalised strength of the modulation potential, defined as the peak-to-peak modulation potential U_{pp} divided by the characteristic kinetic energy E_K. Here, $E_K = g^2/2m$ for GaAs and $E_K = v_F g$ for graphene, where g is the reciprocal lattice vector of the artificial crystal. Our system uniquely enables access to a previously unexplored regime, where the modulation is strong ($U_{pp} \gg E_K$) and the Fermi energy can be continuously tuned across multiple bands.

II. MEASUREMENTS IN THE WEAK MODULATION REGIME

To access the very weak modulation regime, the superlattice potential strength must be reduced by using a deeper heterostructure. The deeper the heterostructure, the weaker the superlattice potential. The effect of the wafer depth on superlattice potential strength is discussed in Supplementary section III. Here we present measurements on a device fabricated with a 37 nm deep heterostructure (W916) and demonstrate how it can be continuously tuned from a plain 2DEG to an artificial crystal.

In the very weak modulation regime ($V_{TG} = +0.5$ V), the system behaves like a conventional 2D electron gas (2DEG), as indicated by the featureless Hall resistance near $B = 0$ in Fig. S2(a),(d) and a negative Hall coefficient in Fig. S2(g). The Hall coefficient is inversely proportional to the

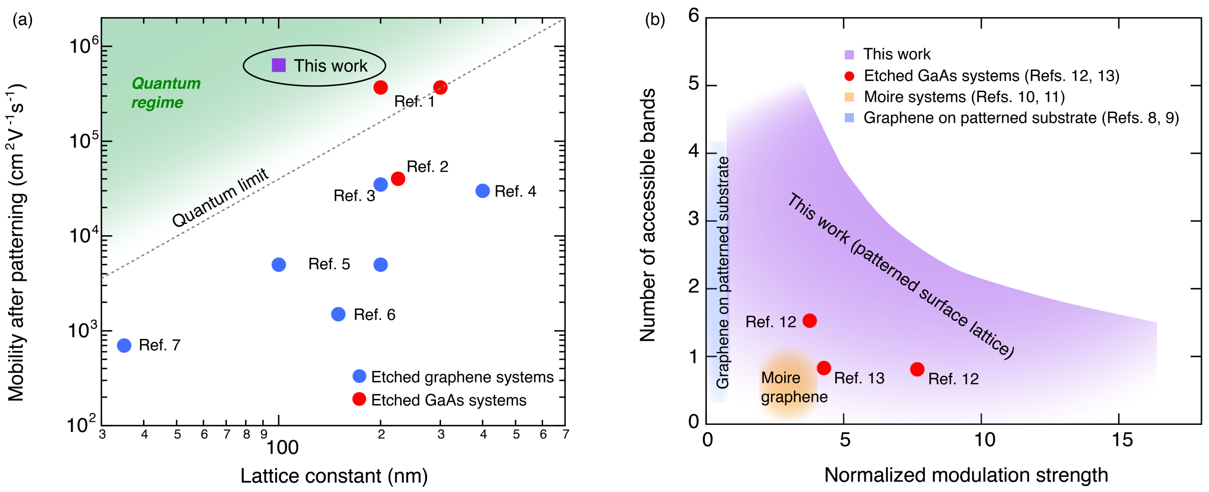

FIG. S1. (a) Carrier mobility of the patterned system versus the artificial lattice constant for etched systems with a strong modulation potential. Red circles represent etched GaAs heterostructures (Refs. 1 and 2), blue circles represent etched graphene systems (Refs. 3–7) and the violet square represents our work. The green shaded region denotes the quantum regime, where the mean free path exceeds the artificial lattice constant, allowing new bandstructure effects to be observed. The dashed line marks the boundary between the classical and quantum regimes, where the two length scales are equal. (b) The number of accessible mini-bands versus the normalized modulation potential strength U_{pp}/E_K. The shaded blue area corresponds to graphene on a patterned substrate (Refs. 8 and 9), the shaded orange area represents graphene moiré systems (Refs. 10 and 11), and the red dots represent etched GaAs heterostructures (Refs. 12 and 13). The shaded violet area indicates the regime accessible in the present work, where artificial bands are clearly resolved, with potential modulation much larger than the kinetic energy, while the Fermi energy can be continuously tuned through multiple artificial bands.

2DEG density $n = 1/(edR_{xy}/dB)$, which is directly proportional to the patterned gate voltage V_{PG}.

When the modulation potential is increased, by setting the top-gate voltage to $V_{TG} = -0.5$ V, we observe suppression of the Hall resistance around $B = 0$ (Fig. S2(b)). Further increasing the potential modulation to $V_{TG} = -1$ V causes the Hall resistance around $B = 0$ to reverse, resulting in a positive Hall slope (Fig. S2(c,f)). This sign reversal of the slope is reflected in the Hall coefficient, highlighted by the red-coloured region in Fig. S2(i), providing direct evidence of hole-like carriers.

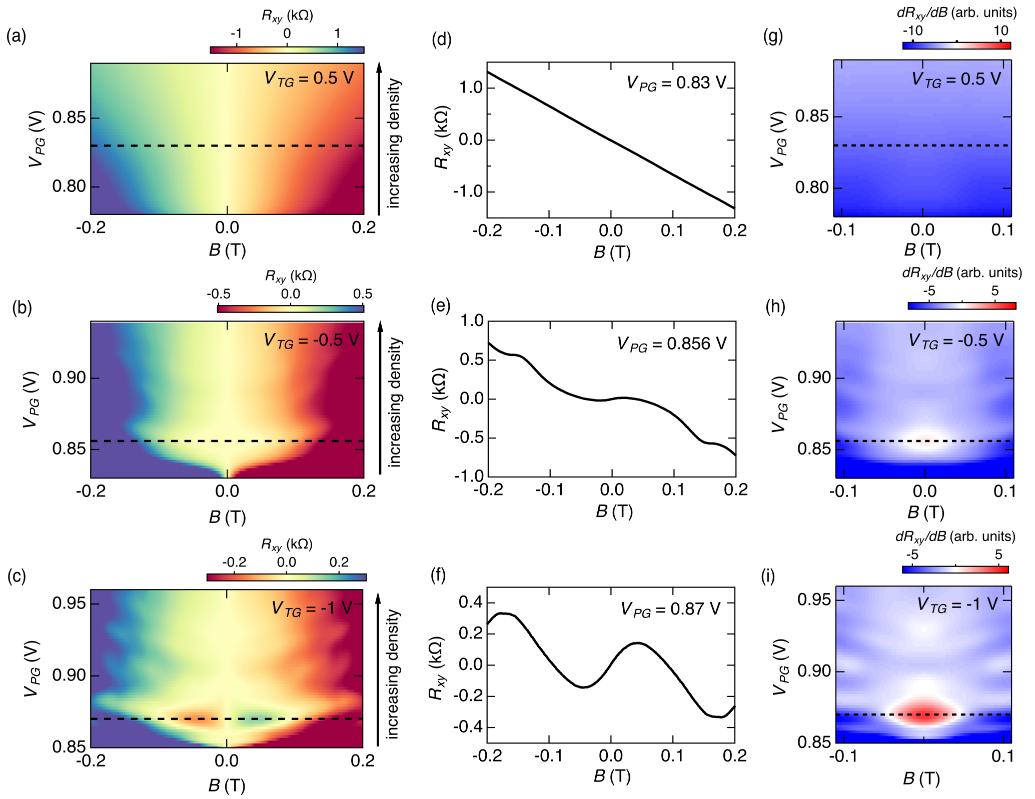

FIG. S2. Hall measurements of device D37 on the 37 nm deep heterostructure W916 with a lattice constant of 120 nm at $T = 1.5$ K. The first column shows the evolution of the Hall resistance as a function of the patterned gate voltage V_{PG} and perpendicular magnetic field B at increasing modulation strengths: (a) $V_{TG} = 0.5$ V, (b) $V_{TG} = -0.5$ V and (c) $V_{TG} = -1$ V. Dashed lines indicate the position of the linecuts for the second column. The second column (d)-(f) shows the Hall resistance as a function of B at (d) $V_{PG} = 0.83$ V, (e) $V_{PG} = 0.858$ V and (f) $V_{PG} = 0.87$ V corresponding to the three modulation strengths. These panels demonstrate the change in Hall resistance near zero-field as the modulation strength increases. The third column (g)-(i) shows the Hall coefficient $R_H = dR_{xy}/dB$ as a function of V_{PG} and B at the three modulation strengths corresponding to panels (a)-(c). Red regions indicate where the Hall coefficient becomes positive, signifying a transition to hole-like carriers.

III. BANDSTRUCTURE EVOLUTION AND CALIBRATION OF THE FILLING FACTOR

In Fig. S3 we present the measured Hall coefficient R_H for different modulation strengths. Band filling can be determined by tracking the positions of the van Hove singularities(VHs) and Dirac points (DPs) where R_H changes sign. As the modulation strength gradually increases with a more negative top-gate voltage, the mini-bands shift apart and become more distinct, leading to additional sign changes at higher densities (higher V_{PG}). The size of one band in gate voltage,

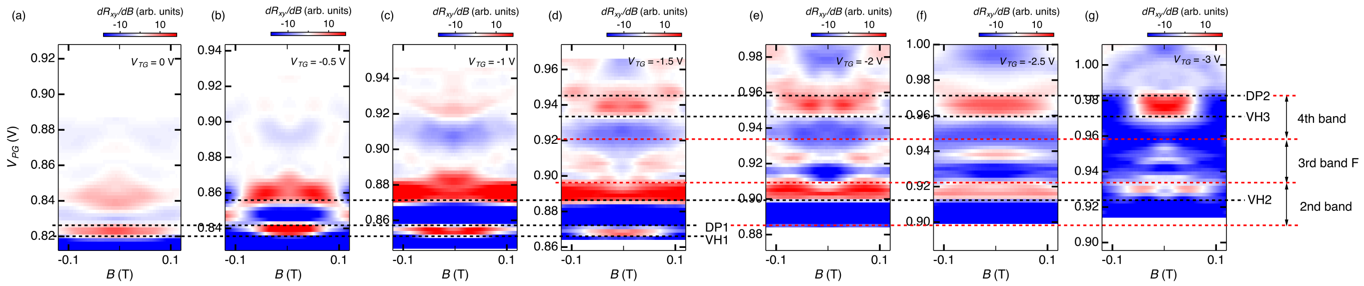

FIG. S3. Measured Hall Coefficient $R_H = dR_{xy}/dB$ as a function of patterned gate voltage V_{PG} and perpendicular magnetic field $B_\perp$ for device D251 on 25 nm deep heterostructure W1740 with a lattice constant of 100 nm at $T = 1.5$ K. The modulation strength is gradually increased by stepping the top-gate voltage V_{TG} as follows: (a) $V_{TG} = 0$ V, (b) $V_{TG} = -0.5$ V, (c) $V_{TG} = -1$ V, (d) $V_{TG} = -1.5$ V, (e) $V_{TG} = -2$ V, (f) $V_{TG} = -2.5$ V and (g) $V_{TG} = -3$ V. Black dashed lines indicate the positions of the Dirac points (DP1 and DP2) and van Hove singularities (VH1, VH2, VH3) where Hall slope changes sign. Red dashed lines mark the positions where each band terminates. Since density is linearly proportional to V_{PG}, the red dashed lines are evenly spaced, with a band size of $\Delta V_{PG} \approx 24$ meV.

ΔV_{PG}, can be extracted in two ways: (1) from the first Dirac point (DP1) to the end of the hole-like (red) region at the second band edge, corresponding to filling one band (Fig. S3(d)); or (2) from the end of this hole-like (red) region to the second Dirac point (DP2), corresponding to filling two band (Fig. S3(f)-(g)). Using these methods, we extract $\Delta V_{PG} \approx 24$ mV, which for a triangular lattice with $a = 100$ nm, corresponds to a density of $n_0 = 2.3 \times 10^{10}$ cm^{-2}. A fully occupied band corresponds to two electrons per band due to spin degeneracy, establishing a direct conversion between ΔV_{PG} and the electron filling factor ν. Specifically, each 24 mV change in V_{PG} corresponds to additional two electrons per unit cell. This conversion factor depends solely on the wafer depth (assuming a fixed lattice constant), as the device can be approximated as a parallel plate capacitor (see supplementary section XI).

There are two key subtleties in the band assignment. First, at an intermediate modulation strength (Fig. S3(b)-(c)) the end of the second band can overlap with the third band (Fig. 1(d)). In this case, a negative Hall coefficient can still appear due to the coexistence of electron-like and hole-like carriers. This explains why the positive Hall slope region above the second van Hove singularity (VH2) spans over a larger ΔV_{PG} than expected (Fig. S3(a)-(c)). When the second and the third band are fully separated, the width of the positive Hall coefficient region above the first Dirac point (DP1) can be used to calibrate the gate voltage corresponding to one band filling. Second, as the modulation strength increases (Fig. S3(e)-(g)), transport in the first band gradually

diminishes, and the sign changes corresponding to the graphene-like bands becomes suppressed. This is possibly due to disorder in modulation potential and localization of electrons in the strong modulation regime. However, by tracking the evolution, we can still identify Hall resistance features of the hole-like region at the second band edge, providing a reliable reference point in the strong modulation regime.

In Fig. S4 we show the bandstructure evolution of another device, D33, fabricated on a different wafer with a 33nm deep heterostructure and a 100 nm lattice constant. Its behaviour closely resembles that of D251, except for a slightly weaker modulation strength. The strength of the modulation potential is predominantly determined by two experimental factors related to the device architecture: (1) the distance between the patterned gate and the 2DEG d and (2) the lattice constant a (see Ref. 14). The modulation strength increases exponentially as d decreases. Increasing a also enhances the modulation but reduces the band density, making it harder to observe mini-bands. The optimal lattice constant is $\sim$ 100 nm, corresponding to a band density of $\sim 2 \times 10^{10}$ cm^{-2}, which is readily achievable in GaAs/AlGaAs heterostructures.

A larger d also decreases the capacitance of the patterned gate, which, for the same lattice constant and band density, proportionally increases the band size ΔV_{PG}. From the 33nm deep heterostructure we extract a band filling of $\Delta V_{PG} \approx 32$ mV. The change in ΔV_{PG} per band compared to the 25nm deep heterostructure (Fig. S3, $\Delta V_{PG} = 24$ mV) precisely reflects the change in depth assuming a simple parallel plate capacitor, where $\Delta V_{PG}/d \propto n_0 = 2.3 \times 10^{10}\ cm^{-2}$.

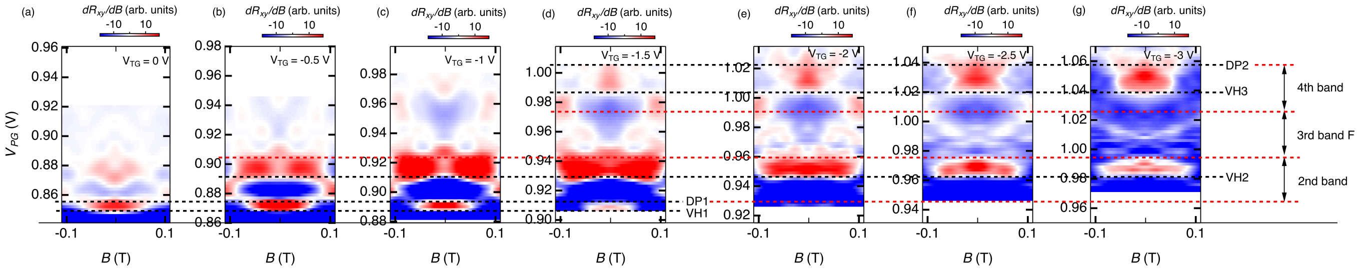

FIG. S4. Hall Coefficient $R_H = dR_{xy}/dB$ as a function of patterned gate voltage V_{PG} and perpendicular magnetic field $B_\perp$ for device D33 on the 33 nm deep heterostructure W1735 with a lattice constant of 100 nm at $T = 1.5$ K. The modulation strength is gradually increased by stepping the top-gate voltage V_{TG} as follows: (a) $V_{TG} = 0$ V, (b) $V_{TG} = -0.5$ V, (c) $V_{TG} = -1$ V, (d) $V_{TG} = -1.5$ V, (e) $V_{TG} = -2$ V, (f) $V_{TG} = -2.5$ V and (g) $V_{TG} = -3$ V. Black dashed lines indicate the positions of the Dirac points (DP1 and DP2) and van Hove singularities (VH1, VH2, VH3) where Hall slope changes sign. Red dashed lines mark the positions where each band terminates. Since density is linearly proportional to V_{PG}, the red dashed lines are evenly spaced, with a band size of $\Delta V_{PG} \approx 32$ meV.

IV. REPRODUCIBILITY ACROSS MULTIPLE DEVICES

We have measured multiple devices across three different heterostructures: W1740 which is 25 nm deep (Figs in the main text and Fig.S3, S5, S6, S7), W1735 which is 33 nm deep (Fig.S4) and W916, which is 37 nm deep (Fig.S2), as detailed in Table I. The devices on the 25 nm and 33 nm deep wafers were fabricated with a 100 nm lattice while the device on the 37 nm deep heterostructure has a 120 nm lattice. Despite differences in the accessible range of modulation strength across different wafers, we observe very similar behaviour in all devices.

Device Name	Wafer Number	Wafer Depth	Lattice Constant	Device Geometry
D251	W1740	25 nm	100 nm	Van der Pauw, 5μm$\times$5μm
D252	W1740	25 nm	100 nm	Hallbar, 2μm wide channel
D253	W1740	25 nm	100 nm	Hallbar, 2μm wide channel
D254	W1740	25 nm	100 nm	Hallbar, 3μm wide channel
D37	W916	37 nm	120 nm	Van der Pauw, 10μm$\times$10μm
D33	W1735	33 nm	100 nm	Van der Pauw, 5μm$\times$5μm

TABLE I. List of devices.

On the shallowest heterostructure (W1740, 25 nm deep) where the modulation strength is the strongest, we have measured multiple devices: D251 (Van der Pauw geometry), D252 (Hall bar geometry, 2 μm wide channel), D253 (Hall bar geometry, 2 μm wide channel), and D254 (Hall bar geometry, 3 μm wide channel). In Fig. S5(a-c) (D252), Fig S6 (D253) and Fig. S7 (D254), we show the measured Hall resistance R_{xy}, Hall coefficient $R_H = dR_{xy}/dB$, and longitudinal resistance R_{xx} at $V_{TG} = -1.5$ V where the band assignment is performed, for 3 different devices respectively. All devices exhibit the same band size $\Delta V_{PG} \approx 24$ mV, as expected, with a resistance peak at the centre of the flat band.

In Fig. S5(d-f), we also show measurements at a stronger modulation ($V_{TG} = -2$ V) for device D252, which corresponds to the configuration used in Fig. 4 of the main text. Due to the strong modulation effects on the Hall signal at low densities, band assignment here relies on the anchor point DP2. Once the position of DP2 is determined, the position of the flat band is known, as the band size is 24 mV. We estimate the error in determining the position of DP2 to be $\lesssim 2$ mV, as highlighted by the grey shaded area in Fig. S5(d-f), which corresponds to 8% of the band density.

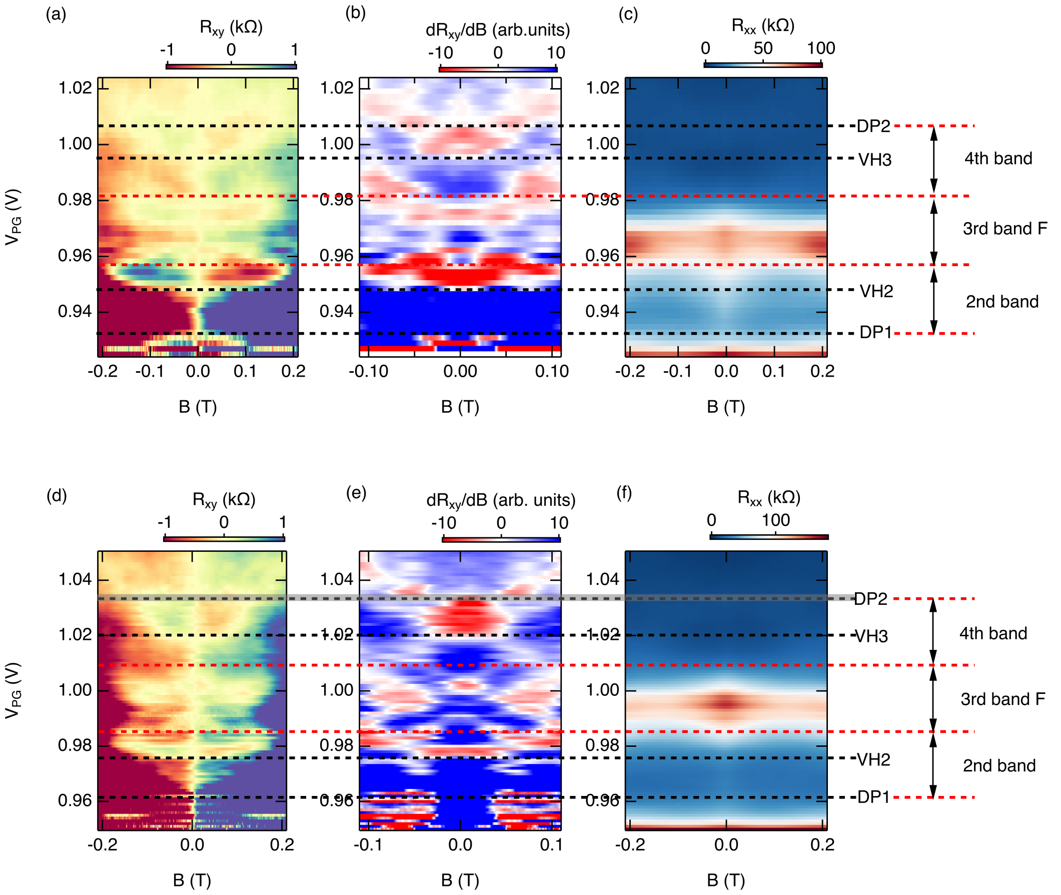

FIG. S5. Hall resistance R_{xy}, Hall coefficient R_H and longitudinal resistance R_{xx} as a function of patterned gate voltage V_{PG} and perpendicular magnetic field $B_\perp$ for device D252 on the 25 nm deep heterostructure W1740 with a lattice constant of 100 nm at $T = 1.5$ K. Modulation strength is kept at $V_{TG} = -1.5$ V (a-c) and $V_{TG} = -2$ V (d-f). In both cases, the kagome bands are developed. Black dashed lines indicate the positions of the Dirac points and the van Hove singularities where the Hall slope changes sign. Red dashed lines mark the band filling. The grey shaded area in (d-f) indicates the uncertainty (approximately 8%, or (2 mV) in band assignment when determining the position of the anchor point DP2.

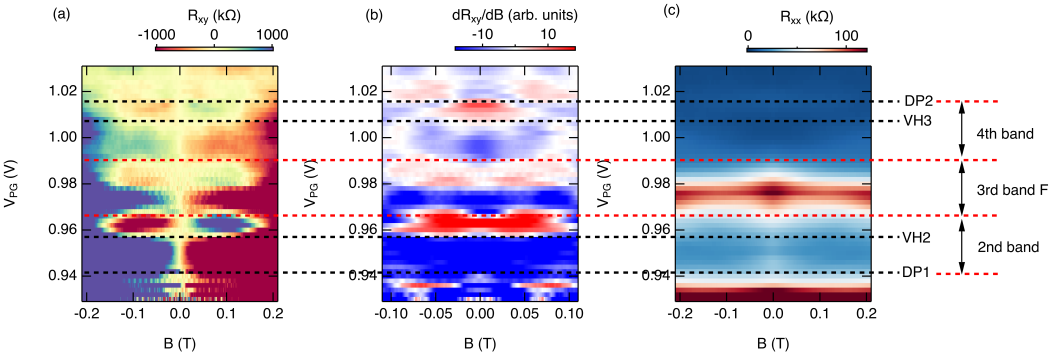

FIG. S6. Hall resistance R_{xy} (a), Hall coefficient R_H (b), and longitudinal resistance R_{xx} (c) as a function of patterned gate voltage V_{PG} and perpendicular magnetic field $B_\perp$ for device D253 on the 25 nm deep heterostructure W1740 with a lattice constant of 100 nm at $T = 1.5$ K. Modulation strength is kept at $V_{TG} = -1.5$ V, where the kagome bands are developed. Black dashed lines indicate the positions of the Dirac points and the van Hove singularities where Hall slope changes sign. Red dashed lines indicate the band filling.

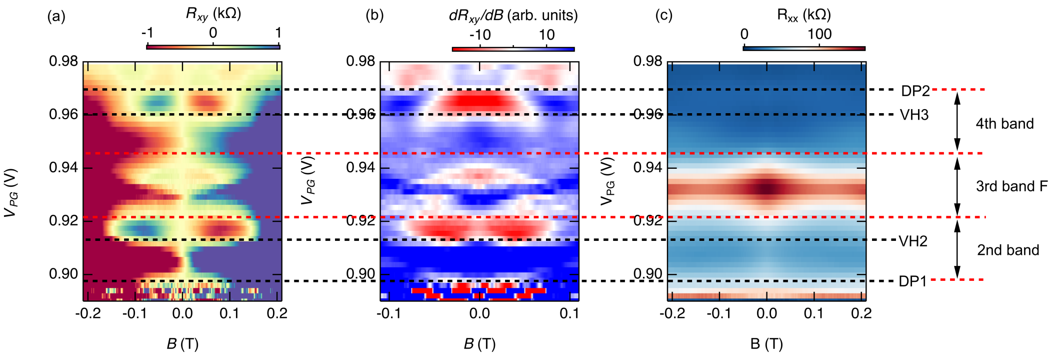

FIG. S7. Hall resistance R_{xy} (a), Hall coefficient R_H (b), and longitudinal resistance R_{xx} (c) as a function of patterned gate voltage V_{PG} and perpendicular magnetic field $B_\perp$ for device D254 on the 25 nm deep heterostructure W1740 with a lattice constant of 100 nm at $T = 1.5$ K. Modulation strength is kept at $V_{TG} = -1.5$ V, where the kagome bands are developed. Black dashed lines indicate the positions of the Dirac points and the van Hove singularities where Hall slope changes sign. Red dashed lines indicate the band filling.

V. BEHAVIOUR OF THE BAND GAP

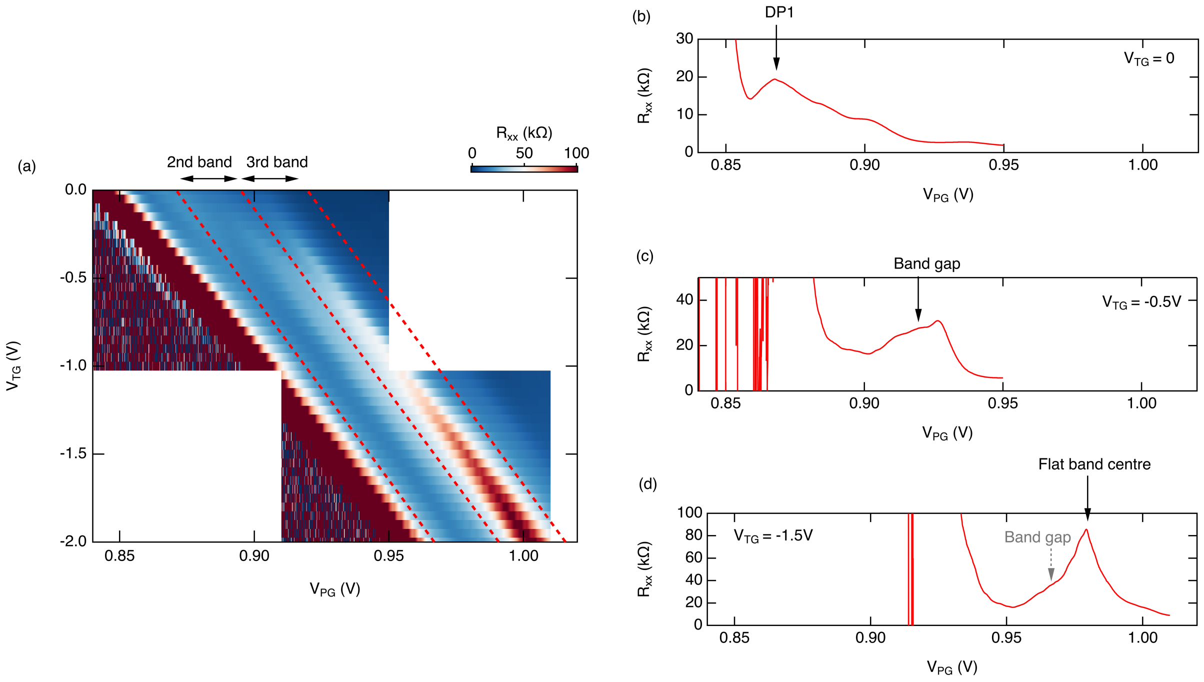

FIG. S8. (a) Longitudinal resistance R_{xx} as a function of patterned gate voltage V_{PG} ($\propto$ density) and modulations strength V_{TG} for device D253 at $T = 1.5$ K. Red dashed lines indicate the band filling. (b-d) Line cuts of R_{xx} at $V_{TG} = 0$, -0.5 V, and -1.5 V, respectively.

In Fig. S8 we show the longitudinal resistance of Device D253 while continuously tuning the modulation strength with no external magnetic field. As V_{TG} becomes more negative (increasing modulation), the band filling corresponding to the same density shifts to higher V_{PG} due to simple capacitive coupling between the two gates.

At different modulation strengths, the strongest R_{xx} peak in the system is generated by different mechanisms. At $V_{TG} = 0$ V (Fig. S8(b)), the resistance peak at the first Dirac point (DP1) is the most resistive. Increasing V_{TG} to -0.5 V results in a broad peak developing near the band gap between the second and third bands (Fig. S8(c)). Finally, at $V_{TG} = -1.5$ V, a strong but broad resistance peak across the flat band dominates, with a resistance much larger than that of either the Dirac peak or the band gap peak (Fig. S8(d)).

A slight resistance rise is still visible in Fig. S8(d), where the position of the band gap is indicated by the grey arrow. However, due to the strong resistance peak at the flat band, features of the band gap becomes less visible once the kagome flat band is fully developed.

VI. MEASUREMENTS OF DIFFERENT CONFIGURATIONS AND TEMPERATURES ON DEVICE D252

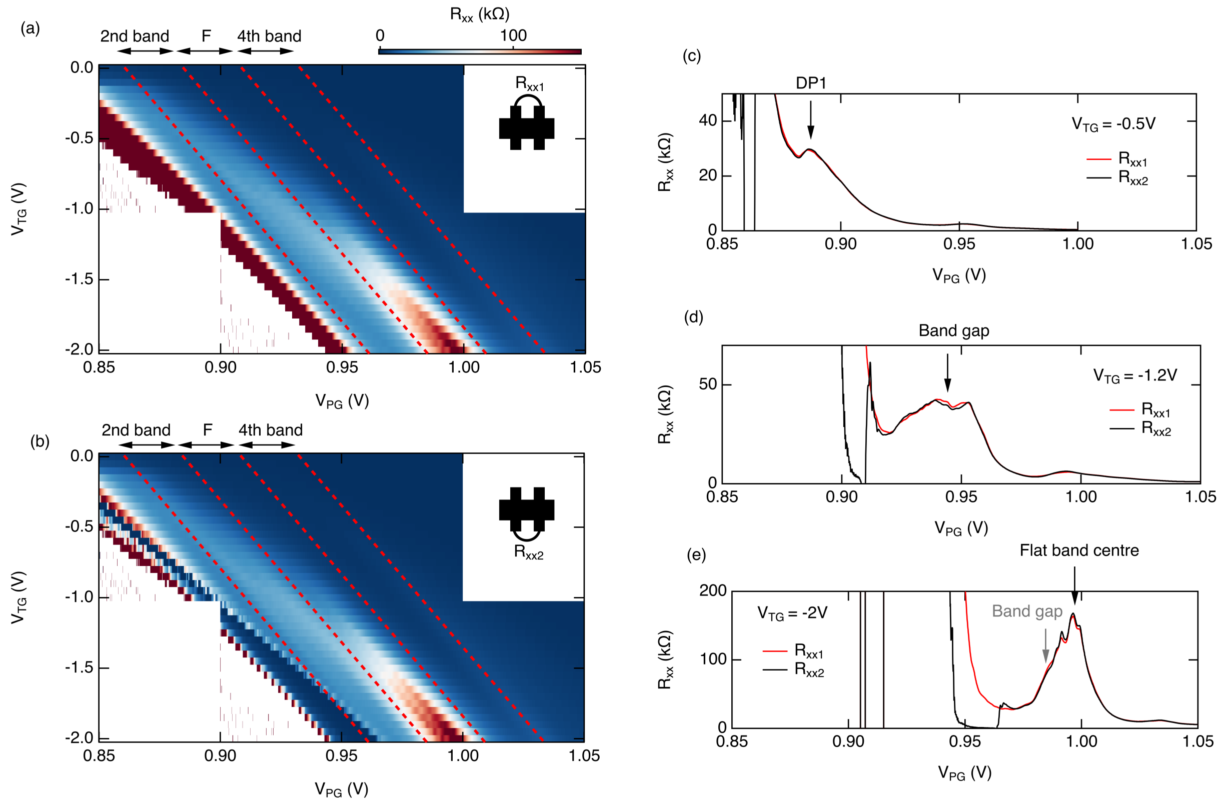

FIG. S9. Longitudinal resistance R_{xx} as a function of patterned gate voltage V_{PG} ($\propto$ density) and modulations strength V_{TG} for device D252 at $T = 1.5$ K in two configurations R_{xx1} (a) and R_{xx2} (b), as illustrated in the insets. In both configurations, the distance between the contact leads is 2μm. Red dashed lines indicate the band filling. (c-e) Line cuts of R_{xx} at $V_{TG} = -0.5$, -1.2 V, and -2 V, respectively.

In this section we first present the results from a second R_{xx} configuration on device D252 as a supplement to the results shown in the main text. As illustrated in the schematics in Fig. S9, R_{xx} can be measured using two different pairs of contacts on opposite sides of the Hall bar. In the main text (Fig.4), R_{xx1} is presented. In Fig. S9, we show both R_{xx1} and R_{xx2} while continuously tuning the modulation strength via V_{TG}. The same behaviour of the longitudinal resistance as observed in Device D253 (Section V) is seen in both configurations. The only discrepancy between the two configurations is that contacts used for R_{xx2} stop working at low densities earlier than those used for R_{xx1}. This is indicated by a zero resistance in R_{xx2} near pinch-off.

In Fig. S10 we show the same measurement as in Fig.4 for configuration R_{xx2}. As with the previous configuration, the zero resistance at low densities ($\nu < 3$) is attributed to the failure of

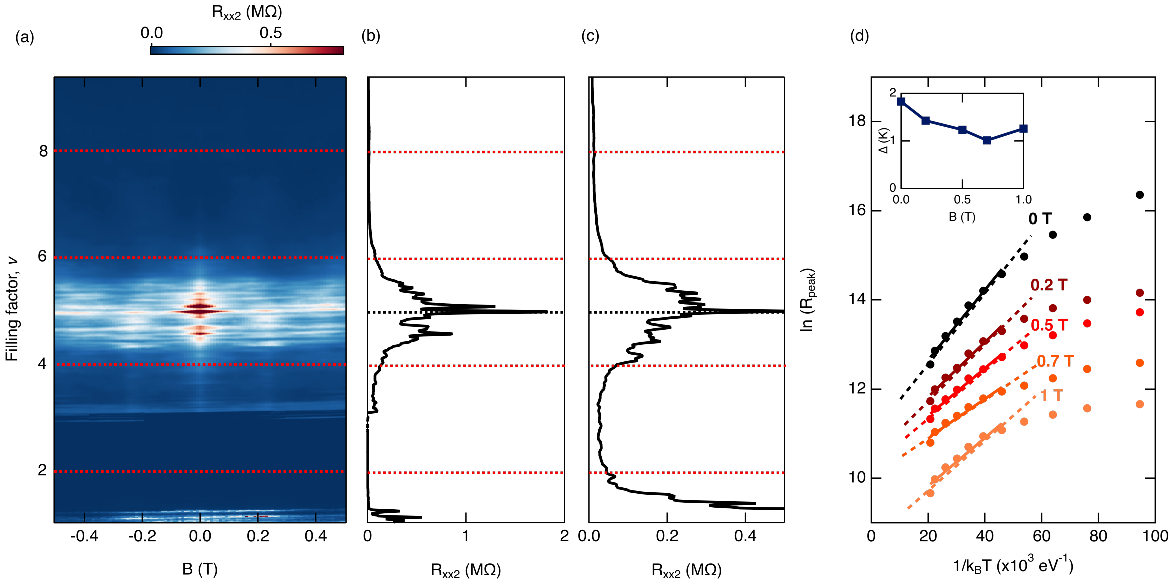

FIG. S10. (a) Measured longitudinal resistance of device D252 with $V_{TG} = -2$ V at $T = 350$ mK in configuration R_{xx2}. Red dashed lines indicate the band filling. Line cuts of R_{xx2} at different magnetic fields: $B_\perp = 0$ (b) and $B_\perp = 0.1$ T (c), with black dashed lines indicating the half filling of the flat band. (d) Arrhenius plot (circles) of the resistance of the half-filling insulating state at different $B_\perp$. The solid lines (with dashed extrapolation) show the fitting with $\exp\left[-\Delta/(2k_BT)\right]$. The inset shows the extracted thermal activation gap Δ as a function of $B_\perp$.

ohmic contacts. However, this does not affect the behaviour of the device in the flat band, which is our focus. A sharp resistance peak at half filling of the flat band is also observed in R_{xx2}, with a thermal activation gap of $1-2$ K, similar to R_{xx1}.

In Fig. S11, we show the measured longitudinal resistance for both configurations at the base temperature of the dilution refrigerator, with an estimated electron temperature of ~ 100 mK. The observed behaviour, including the resistance peak at the centre of the flat band and its field response, is qualitatively similar to that shown in Fig.4 of the main text, where $T = 350$ mK. However, the magnitude of the resistance peak at $T \sim 100$ mK is approximately an order of magnitude higher.

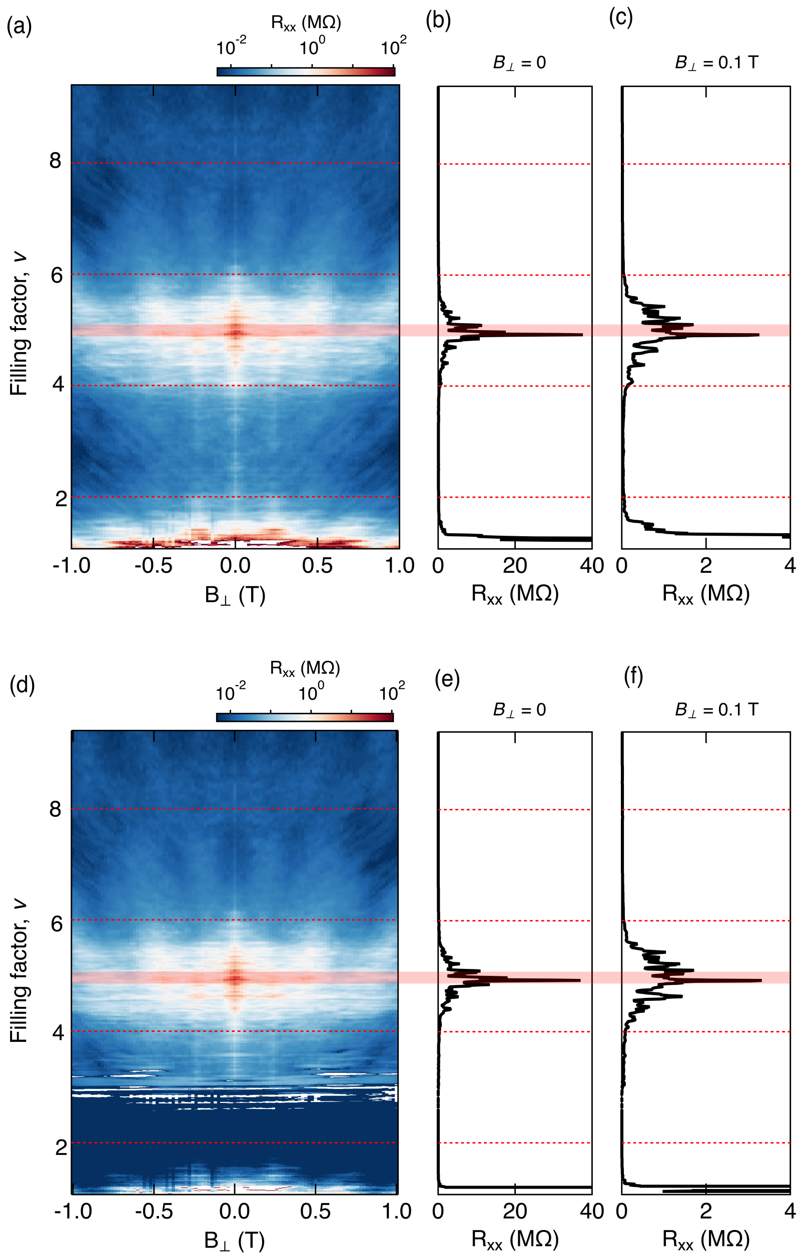

FIG. S11. Measured longitudinal resistance R_{xx} of device D252 at $V_{TG} = -2$ V and $T \sim 100$ mK, along with the corresponding line cuts at $B = 0$ and $B = 0.1$ T. (a)-(c) Measurement obtained using configuration R_{xx1} (same as Fig.4 in the main text). (d)-(f) Measurement obtained using configuration R_{xx2} (same as Fig. S10).

VII. REPRODUCIBILITY OF THE CORRELATED STATE AND THE ROLE OF DISORDER

In Fig. S12 we present low-temperature measurements of three Hallbar devices D252, D253 and D254, each consistently showing a sharp resistance peak at 1/3 filling of the kagome lattice ($\nu = 5$). The reproducibility of this central resistance peak across multiple devices strongly argues against a disorder-driven single-particle mechanism, which would vary from device to device. Disorder-driven effects, such as mesoscopic fluctuations—arising from random networks of conducting channels due to inhomogeneity-induced non-uniform current flow (i.e. percolation)—would lead to aperiodic magnetoresistance, as observed in universal conductance fluctuations (UCFs) in GaAs, gold and silicon samples[15,16]. In contrast we observe a non-random dependence on magnetic field, with smaller periodic oscillations commensurate with the flux per unit cell as shown in Fig. S13. The consistent observation of the central resistance peak across multiple devices, combined with the absence of disorder-like magnetoresistance signatures, definitively rules out single-particle mesoscopic disorder-related mechanisms as the origin of the central resistance peak. Furthermore, the B-dependence of Rxx at the flat band centre (Fig. S13(b)) is very similar to that of the small satellite peaks (Fig. S13(a)(c)), indicating a common physical origin.

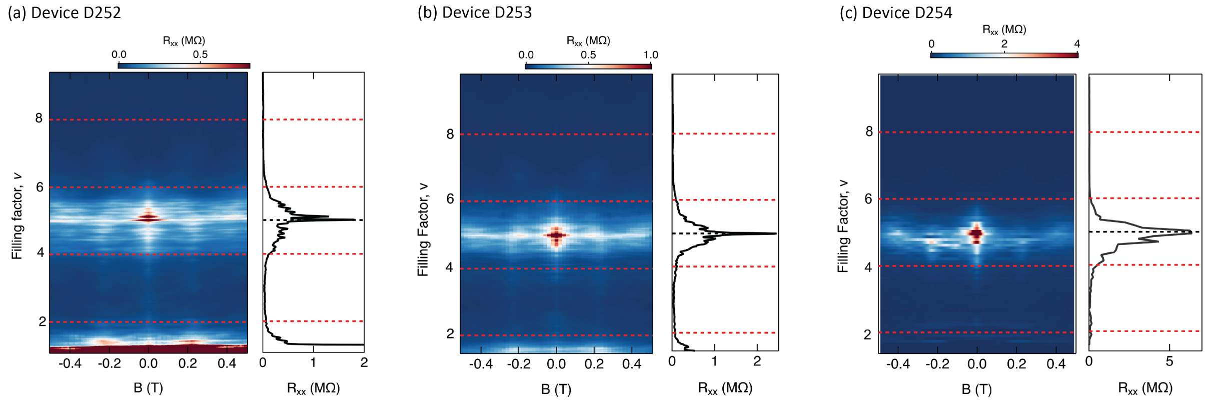

FIG. S12. Low-temperature longitudinal resistance R_{xx} as a function of filling factor ν and perpendicular magnetic field $B_{\perp}$, with linecuts at $B = 0$ for (a) device D252 at $V_{TG} = -2$ V (same data as Fig.4(a) in the main text); (b) device D253 at $V_{TG} = -2$ V; and (c) device D254 at $V_{TG} = -1.5$ V (details about the devices can be found in Table. I). The data in panel (c) has lower resolution in filling factor compared to panels (a) and (b), as it was taken by sweeping $B_{\perp}$ while stepping V_{PG}, whereas in panels (a) and (b), V_{PG} was swept while $B_{\perp}$ was stepped.

Upon increasing temperature, we observe that the sharp resistance at $\nu = 5$, associated with the commensurate Wigner crystal state, progressively weakens as shown in Fig.S14. The peak is signifi-

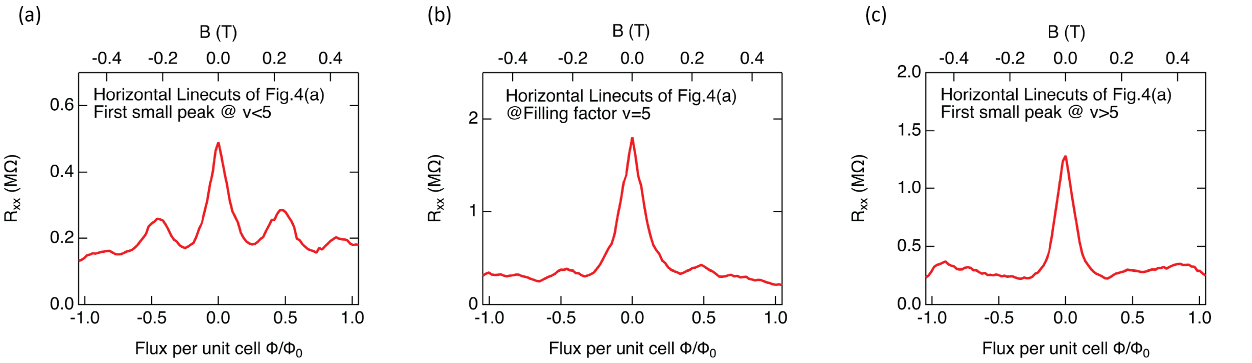

FIG. S13. Horizontal linecuts of Fig.4(a) at the first small satellite peak below $\nu = 5$ (a), $\nu = 5$ (b), and the first small satellite peak above $\nu = 5$ (c) as a function of flux per unit cell on the bottom axis and perpendicular magnetic field on the top axis.

cantly reduced at T=0.6 K and is completely absent by T=1.5 K, leaving only a broad background feature. The disappearance of the sharp resistance peak indicates the loss of long-range charge order of the commensurate Wigner crystal, while the remaining smooth background is consistent with a disordered correlated state within the flat band. Because of phase separation of ^{3}He/^{4}He mixture, the temperature of the dilution fridge becomes unstable when heated above T$\sim$0.6 K, which prevents us from performing detailed studies to determine a precise melting temperature of the commensurate Wigner crystal.

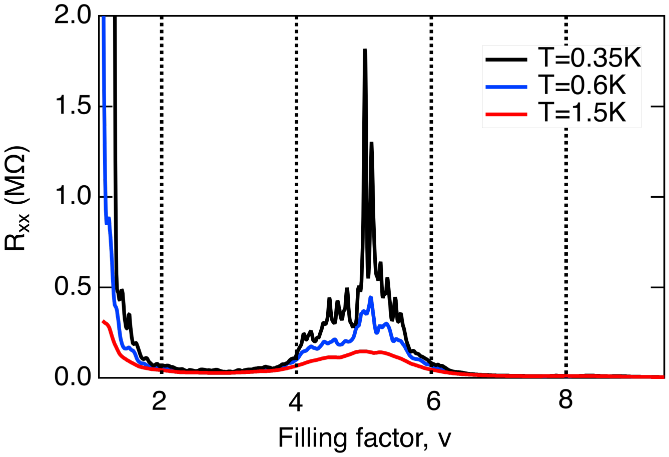

FIG. S14. Measured longitudinal resistance R_{xx} of device D252 at $V_{TG} = -2$ V at three different temperatures: T=0.35K (the same as in Fig.4b), T=0.6K and T=1.5K.

VIII. COULOMB SCREENING, EFFECT ON MODULATION STRENGTH

The actual potential ($\widetilde{U}$) experienced by electrons differs from the applied potential (U_0) because of Coulomb screening. We can account for this difference using the Hartree equation

$$\widetilde{U}_q = U_{0q} - \frac{2\pi e^2}{\varepsilon q} n_q \tag{1}$$

Where n_q is the Fourier transform of the spatially varying particle density of a system described by Eqn.(1) in the main text with $U = \widetilde{U}$. Here $\varepsilon = 13$ is the dielectric constant of GaAs. The subscripts q denote Fourier coefficients. The Hartree equation must be solved at a particular Fermi energy, E_F. In real space, the particle density is defined by

$$n(r) = \sum_{\substack{k,n \\ E_{k,n} < E_F}} |\psi_{k,n}(r)|^2 \tag{2}$$

Where $\psi_{k,n}(r)$ are the Bloch functions obtained via numerical diagonalisation (see Methods). To solve the Hartree equation we assume that $\widetilde{U}$, like U_0, is sinusoidal, meaning that its only non-zero Fourier component is $\widetilde{U}_{g_i}$. Evaluating the Hartree equation at $q = g_i$ we obtain

$$\widetilde{W} = W_0 - \frac{2\pi e^2}{\varepsilon g_i} \frac{n_{g_i}}{A_{cell}} \tag{3}$$

Where A_{cell} is the unit cell area in real space and

$$n_{g_i} = \int_{cell} d^2 r \; n(r) e^{i g_i \cdot r} \tag{4}$$

Using Eqn. 3 we can find the strength of the applied potential W_0 necessary to obtain a self-consistent potential strength $\widetilde{W}$. The results of this calculation are presented in Fig. S15. We remind that the parameter W is related to the peak-to-peak width U_{p-p} of the potential by $U_{p-p} = 9W$.

There are two consequences of the Hartree self-consistency: (i) change to the amplitude W_0 to the self-consistent one, $W_0 \to \widetilde{W}$, plotted in Fig. S15; (ii) appearance of higher harmonics in the potential. The effect (ii) is very weak and can be neglected, see Ref. 14. The effect (i) is significant, but it is fully accounted for since we extract W from the experimental data (the point at which we lose bandstructure effects, as described in section IX). We do not explicitly use Fig. S15, it just

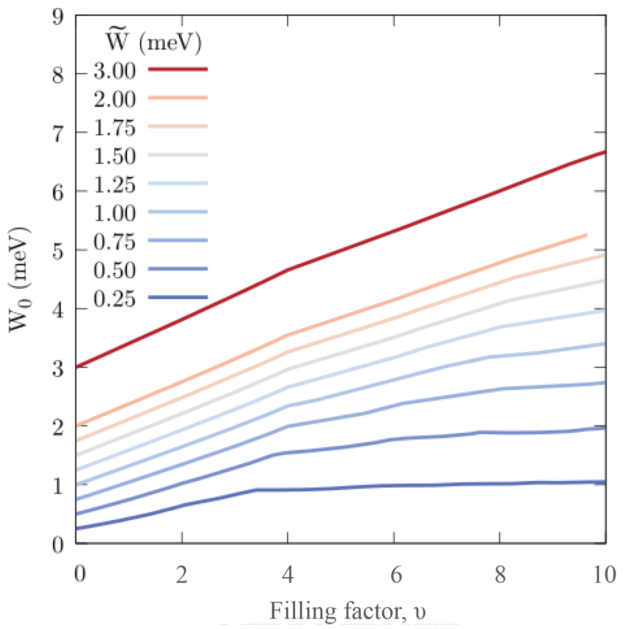

FIG. S15. Effect of Coulomb screening on the potential amplitude. Each curve is the applied potential W_0 necessary to obtain a given self-consistent potential $\widetilde{W}$ at different electron densities. Electron density is given on the horizontal axis in units of the number of filled electron bands. Each curve corresponds to a different value of $\widetilde{W}$.

demonstrates that Hartree screening can change the potential by a factor of 2-4 depending on the density.

IX. ESTIMATION OF MODULATION STRENGTH

Before we can estimate the potential amplitude a few points must first be made clear.

1. Potential amplitude decreases as electron density increases. For example, an imposed potential of $W_0 = 3$ meV is reduced to $\widetilde{W} = 1$ meV when four energy bands are fully filled (at $n = 4n_0 = 9.2 \times 10^{10}$ cm^{-2}, $\nu = 8$). This is the result of screening (Fig. S15).

2. At large potential amplitudes each energy band is distinct, there is no overlap between bands. As the potential amplitude is reduced bands begin to overlap starting with the higher-energy bands first (see Figs. 1c-e in the main text).

3. If the bands of interest (the graphene-like and kagome-like bands) are distinct for a given imposed potential, W_0, then there will be a 'critical' density n_c such that for all $n > n_c$ the

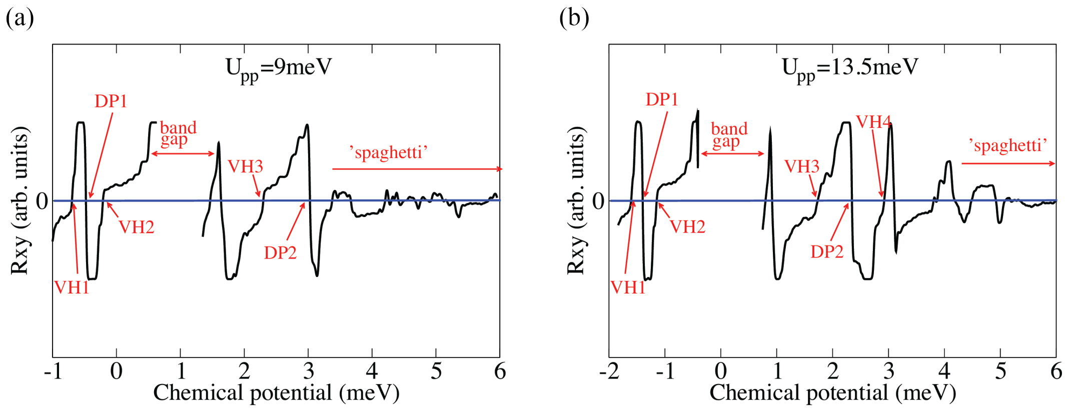

FIG. S16. Numerical KWANT simulations of the Hall resistance with a weak short-range disorder in an artificial lattice for two different modulation potentials (a) $U_{pp} = 9$ meV and (b) $U_{pp} = 13.5$ meV.

energy bands are overlapping. This occurs because the potential strength decreases with density.

4. When the energy bands are distinct there is only ever one kind of charge carrier present for a given electron density (either electron-like or hole-like). When the energy bands overlap both kinds of charge carrier can exist simultaneously. In the latter case no clear signature of the bands is expected in the Hall slope and experimentally this is signaled by a suppression of the Hall response. This is also demonstrated by the KWANT simulation as shown in Fig. S16.

We use these points to estimate the size of the potential amplitude. In particular, we identify the value of V_{PG} which corresponds to n_c. At voltages larger than this V_{PG} no Hall slope transitions are observed. We assign a value of $\widetilde{W}$ at this V_{PG} using our band structure calculation: the band structure must give the correct number of Hall slope transitions below n_c.

Starting with Fig. S3(b) we note that three transitions in the Hall slope are observed below $V_{PG} = 0.87$ V. Above $V_{PG} = 0.87$ V there are no further transitions. We identify the point $V_{PG} = 0.87$ V with the density n_c defined above. Using the notation defined in this section the band structure of Fig. 2(e) was computed for $\widetilde{W}(n_c) = 0.45$ meV. This band structure is consistent with the observed Hall slope transitions and with our estimation that the electron density is less than $n_0/2 = 1/A_{cell} = 1.15 \times 10^{10}$ cm^{-2} at the lowest value of V_{PG} in Fig. S3(b). Using Fig. S15 we find that the imposed potential amplitude must be $W_0 = 1$ meV.

We can repeat this analysis for the full set of maps in Fig.S3. If we consider the strongest modulation Fig. S3(g) the lowest value of V_{PG} corresponds to an electron density just above the first

Dirac point. The first transition above this point corresponds to the second van Hove singularity ($V_{PG} = 0.925$ V). The next transition after this ($V_{PG} = 0.935$ V) is the top of the graphene-like bands. From the second transition point, the subsequent 0.024 V is identified with the third energy band (the flat band). Above the third band there are two further transitions in Hall slope. Again, the difference, ΔV_{PG}, between the top of the flat band and the second subsequent transition is 0.024 V. Each of these transitions, and the spacings between them are consistent with a potential amplitude $\widetilde{W} = 1$ meV at complete filling of four energy bands. At lower fillings the value of $\widetilde{W}$ is larger. Using Fig. S15 we find that $\widetilde{W}$ is 1.25 meV when three bands are fully filled and 1.75 meV when 2 bands are fully filled. Finally, the value of the imposed potential amplitude is $W_0 = 3$ meV. Note that in Fig. 3(b) we plot the bandstructure for $\widetilde{W} = 1.5$ meV ($U_{p-p} = 13.5$ meV) which corresponds to a filling in the flat band region.

X. ESTIMATION OF DISORDER AND MOBILITY

There are 3 kinds of disorder in our device. (i) A short-range disorder related to the presence of impurities in the host semiconductor. (ii) A short-range disorder related to imperfections in nano-lithography process. (iii) A long-range disorder (puddles) related to imperfections in nano-lithography process.

Wafer Number	Wafer Depth (nm)	Mobility (cm^2/Vs)	@Density (cm^{-2})
W916	37	1,000,000	1.8×10^{11}
W1735	33	700,000	1.5×10^{11}
W1740	25	500,000	2×10^{11}

TABLE II. List of wafers.

The disorder of kind (i) can be neglected because the host semiconductor is very clean, with mobilities of the wafers used shown in Table II. Even though we observe that scattering from surface charges becomes increasingly significant and reduces the mobility as the wafer becomes shallower, this is not the dominant source of disorder in our system. Disorder of kinds (ii) and (iii) have been simulated numerically in detail in Ref. 14. It was shown that suppression of short wavelength harmonics due to the Poisson equation make type (ii) less important; puddle formation (type iii) is the most dangerous one, with the size of the puddles ranging from a few to several lattice periods. We thus concentrate on type (iii). First, we assume that puddles result in a Lorentzian broadening of the chemical potential

$$\frac{\Gamma/2}{\pi(\delta\mu^2 + \Gamma^2/4)} \ , \tag{5}$$

Using the width of the resistance peak at the first Dirac point DP1 we estimate the full-width-half-maximum Γ. Fig. S17 shows this peak at two temperatures temperatures, $T = 550$ mK and $T = 1.5$ K. The $T = 1.5$ K peak is slightly broader due to additional temperature broadening. To estimate the disorder broadening Γ we use the $T = 550$ mK data. If we take the base of the peak to be the resistance values at VH1 and VH2 then the full-width-half-maximum at this temperature is $\delta V_{PG} = 7.9$ mV. The difference in V_{PG} between VH1 and VH2 is 16 mV. Thus, the full-width-half-maximum δV_{PG} accounts for half of the change in electron density between VH1 and VH2. To translate this to an energy scale we take half of the energy difference between VH1 and VH2 in the computed bandstructure of Fig. 2d. Doing this we find

$$\Gamma = 0.2 \ meV \tag{6}$$

Next, mobility is estimated using the standard equation

$$\mu = \frac{1}{n_{eff} e \rho_{xx}} \tag{7}$$

Where n_{eff} is the effective density of charge carriers and ρ_{xx} is resistivity. Note that both the chemical potential and the mobility are denoted by the same letter μ, we hope that the meaning of μ will be clear from its context. To estimate mobility near van Hove singularities VH1 and VH2 we have to remember that the effective density at the Dirac point is zero and the effective density at the van Hove singularities is about $n_e \approx n_0/4 = 5.7 \times 10^9$ cm^{-2}. The measured longitudinal resistance presented in Fig. 2(g) and in Fig. S17 is about 2400 Ω; to obtain resistivity we must multiply by the Van der Pauw constant $\frac{\pi}{\ln 2} = 4.53$, appropriate for a device with Van der Pauw geometry. Hence, $\rho_{xx} = 4.53 R_{xx} \approx 11,000\ \Omega$. The mobility close to VH1 and VH2 is about

$$\mu = 100,000 \text{ cm}^2/\text{Vs} \tag{8}$$

This has to be compared with the mobility of a free electron gas in GaAs at density $n_e = n_{eff} = 5.7 \times 10^9$ cm^{-2} which is about $10,000$ cm^2/Vs.

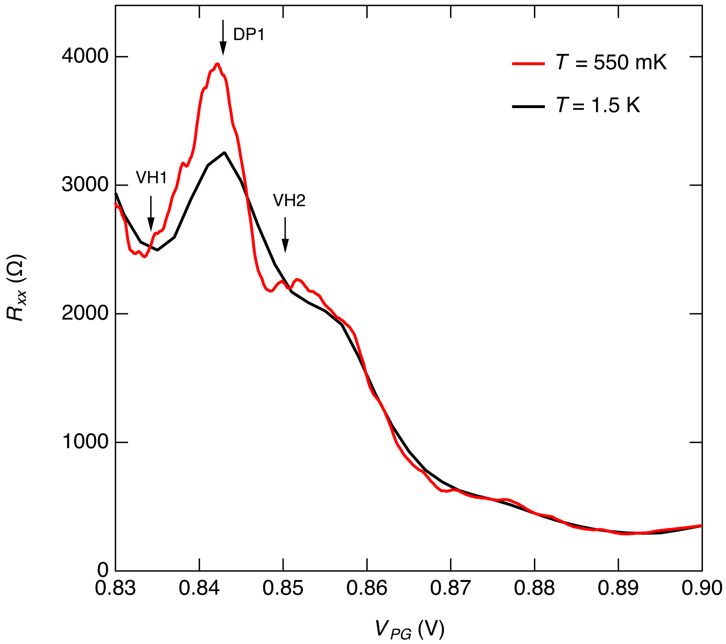

FIG. S17. Measured R_{xx} as a function of V_{PG} at $V_{TG} = -0.5$ V for device D251. Two traces at different temperatures 1.5 K and 550 mK are plotted for comparison. The peak in R_{xx} corresponds to the same Dirac peak in Fig. 2(g) in the main text. Black arrows indicate the positions the Dirac point and van Hove singularities from the Hall measurement.

XI. ESTIMATION OF QUANTUM CAPACITANCE

We consider a simplified device setup with PG and 2DEG as the plates of a parallel plate capacitor. The total energy of the system consists of the energy of electric field $\mathcal{E}_E$ between the plates and energy of electrons $\mathcal{E}_e$ due to the Fermi motion. The energy of electric field comes from the volume between PG and 2DEG

$$\mathcal{E}_E = \frac{\epsilon E^2}{8\pi} A d = 2\pi\sigma^2 A d/\epsilon \tag{9}$$

Here A is the area of the plate and $\sigma = en$ is the density of charge in 2DEG. The value of electric field follows from Gauss law, $E = 4\pi\sigma/\epsilon$. Here we use CGS units. We consider non-interacting electron, hence kinetic energy of all electrons is

$$\mathcal{E}_e = A \int_0^{\mu} \varepsilon \rho(\varepsilon) d\varepsilon \ , \tag{10}$$

where ρ is density of states. Here we set the chemical potential zero at the bottom of the lowest band. Hence, the total energy is

$$\mathcal{E}_t = \mathcal{E}_E + \mathcal{E}_e = 2\pi\sigma^2 A d/\epsilon + A \int_0^{\mu} \varepsilon \rho(\varepsilon) d\varepsilon \ . \tag{11}$$

The variation of energy at the variation of charge, $\delta\sigma = e\delta n$, is

$$\delta\mathcal{E}_t = 4\pi(Ad/\epsilon)\sigma\delta\sigma + A\mu\delta n \tag{12}$$

The chemical potential is determined by the condition

$$n = \int_0^\mu \rho(\varepsilon)d\varepsilon \ . \tag{13}$$

When differentiating Eq.(10) we have in mind that $\frac{\partial}{\partial n} = \frac{\partial\mu}{\partial n}\frac{\partial}{\partial\mu} = \frac{1}{\rho(\mu)}\partial\mu$. This is why the density of states disappears from Eq.(12). To justify the cancellation we need to say that even within a gap there is some small density of states. On the one hand the variation of energy is (12) and on the other hand it is equal to $V\delta Q = VA\delta\sigma$, where is the applied voltage. Hence the relation between voltage and electron number density is

$$V = \frac{4\pi d}{\epsilon}en + \frac{\mu}{e} \tag{14}$$

To find $V(n)$ one needs to solve this equation together with (17).

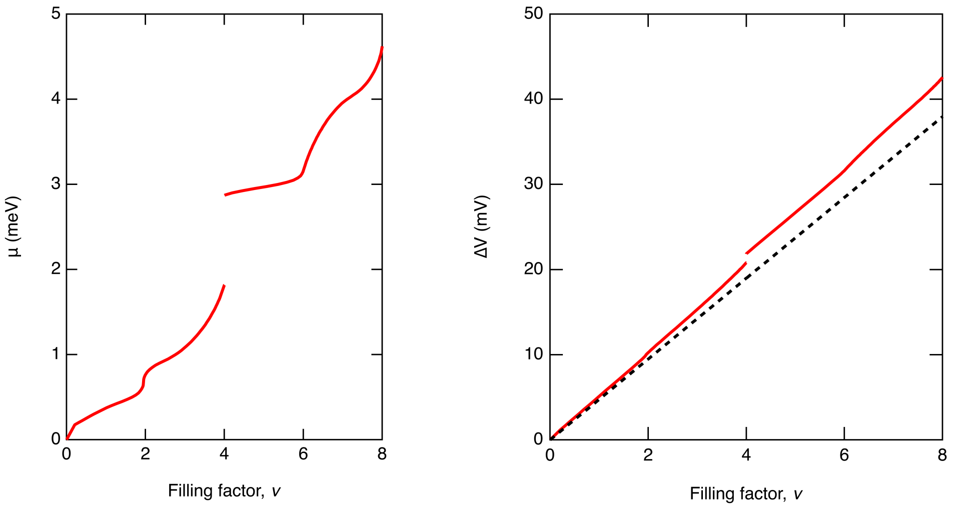

FIG. S18. (a) Chemical potential versus the band filling factor. A single band filling $\Delta\nu = 2$ corresponds to the electron density variation $\Delta n = 2.31 \times 10^{-10} cm^{-2}$. (b) Voltage versus the band filling factor. The red solid line represents the calculation with account of the quantum capacitance. The black dashed line is given by the simple classical formula.

Consider the case of a modulation strength $U_{p-p} = 13.5$meV (Fig.3(c) of main text) and $\mu(n)$ for this dispersion (Fig.S18(a)), we can calculate $V(\nu)$ using Eq. (14). In Fig. S18(b) we plot the calculated $V(\nu)$ (equivalently ΔV_{PG} in experiment) for $d = 25$ nm as the red solid trace. At $\nu = 4$ there is a tiny discontinuity ~ 1 mV due to the band gap. In comparison we also plot the

simple classical case $V = \frac{4\pi d}{\epsilon} en$ (black dashed line). The calculated average slop $\Delta V = 10$meV per $\Delta\nu = 2$ is smaller than the experimental value $\Delta V_{PG} = 24$meV because we use a simplified model of the capacitor. In the real device, the thickness of the 2DEG is ~ 15 nm, which gives the capacitor a much larger effective distance d. The real electric field is also much more complicated because of the superlattice patterning. Nonetheless, this calculation shows that deviations from the straight line due to the quantum capacitance contribution are very small and overall the filling factor is still linear to gate voltage V_{PG}. If considering the real band size of $\Delta V_{PG} = 24$ mV, the disorder-related discontinuity of ~ 1 mV caused by the band gap is $\sim 4\%$ of the band capacity.

XII. LOOP CURRENT WIGNER INSULATOR

We observe the strong insulating behaviour at half filling of the flat kagome band. This filling of the band corresponds to 1/3 filling of the kagome tight binding lattice as it is shown in Fig. S19a, where a blue dot represents one electron. This configuration can be stabilised only by the long-

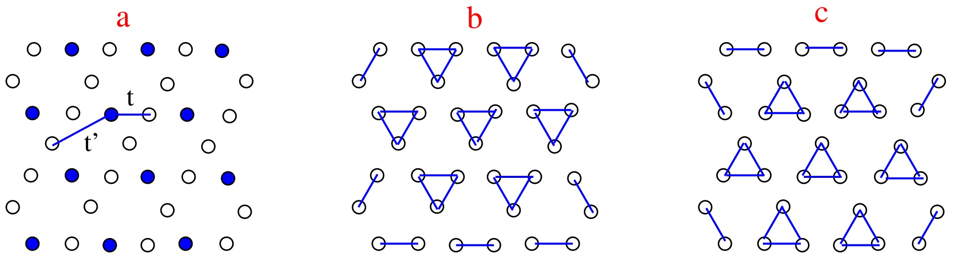

FIG. S19. a. 1/3 filling of the kagome lattice in a commensurate Winger insulator configuration with empty circles representing empty lattice sites and blue circles representing electrons. t and t' indicate the nearest and next nearest neighbour hopping matrix elements respectively. b and c are two possible configurations of the loop current Wigner insulator with solid lines indicating the circulating current.

range Coulomb repulsion. Let us denote by t the nearest site hopping matrix element and by t' the next nearest site hopping matrix element on the kagome lattice (Fig. S19a). The energy of the state Fig. S19a can be lowered by allowing the electron to hop around the triangle as shown in Fig. S19b. The delocalization within the triangle practically does not influence the Coulomb energy, but decreases the kinetic energy. The electron delocalization on a triangle leads to a current around the triangle. This loop current is similar to the loop current suggested for cuprates[17,18]. Hence we arrive at the picture of Loop Current Wigner Insulator that consists of a triangular lattice of loop currents. There are two possible configurations of triangular loop current in a kagome lattice as shown in panels b and c of Fig. S19. Hence, lattice wise, the loop current state is double degenerate.

The electron dispersion in non-interacting approximation has been calculated and we can fit the dispersion using the tight binding model. For the kagome bands the distance between the flat band and the kagome Dirac point (DP2) is $3t$. Hence, comparing with the band structure we conclude that for $U_{p-p} = 13.5$ meV the nearest site hopping matrix element is $t \approx 0.59$ meV and for $U_{p-p} = 27$ meV it is $t \approx 0.53$ meV. Similarly one can fit the next nearest hopping: for $U_{p-p} = 13.5$ meV the value is $t' \approx -0.07$ meV and $U_{p-p} = 27$ meV it is $t' \sim +0.02$ meV. Naturally $t' \ll t$ and t is not very sensitive to U_{p-p} within the reasonable range of the modulation. Hence we neglect t' in all estimates except of the antiferromagnetic interaction of circulating currents which arises only due to t'.

The Coulomb interactions, the on-site Hubbard U, the nearest neighbours interaction V_1 (distance L/2), and the next nearest neighbours interaction V_2 (distance L) are

$$
\begin{aligned}
U &\approx \frac{e^2}{\varepsilon L/4}\left(1-\frac{1}{\sqrt{1+\left(\frac{8d}{L}\right)^2}}\right) \approx 3.5meV \\
V_1 &\approx \frac{e^2}{\varepsilon L/2}\left(1-\frac{1}{\sqrt{1+\left(\frac{4d}{L}\right)^2}}\right) \approx 1.0meV \\
V_2 &\approx \frac{e^2}{\varepsilon L}\left(1-\frac{1}{\sqrt{1+\left(\frac{2d}{L}\right)^2}}\right) \approx 0.2meV
\end{aligned}
\tag{15}
$$

Here $L = 100$ nm is the lattice spacing, $\epsilon = 13$ is the dielectric constant and $d \approx 35$nm is the effective distance to the gate that provides screening via the image charge. V_1 and V_2 are the most important parameters and their estimates are pretty simple and reliable. The estimate for U is rather crude, but it is confirmed by the numerical integration with electron density.

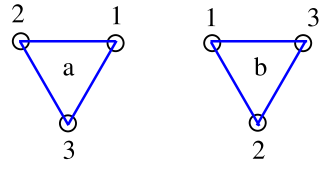

FIG. S20. Two neighbouring triangles a and b of the loop current Wigner insulator with sites on each triangle numbered.

Consider first the single electron dynamics within a triangle. There are three eigenstates

$$\begin{aligned}
\epsilon &= 2t\cos\frac{2\pi}{3} = -t, \qquad a_+^\dagger = \frac{1}{\sqrt{3}}\left[a_1^\dagger + e^{2\pi i/3}a_2^\dagger + e^{4\pi i/3}a_3^\dagger\right] \\
\epsilon &= 2t\cos\left(-\frac{2\pi}{3}\right) = -t, \qquad a_-^\dagger = \frac{1}{\sqrt{3}}\left[a_1^\dagger + e^{-2\pi i/3}a_2^\dagger + e^{-4\pi i/3}a_3^\dagger\right] \\
\epsilon &= 2t\cos(0) = 2t, \qquad a_0^\dagger = \frac{1}{\sqrt{3}}\left[a_1^\dagger + a_2^\dagger + a_3^\dagger\right]
\end{aligned} \tag{16}$$

Here $a_i^\dagger$ is the electron creation operator at the site i of the triangle a. The states for the b-triangle in Fig. S20, $b_+^\dagger, b_-^\dagger, b_0^\dagger$, are similar.

Since $t > 0$ the ground state energy is $-t$, this is the gain in energy due to electron delocalization. The ground state is double degenerate, $a_\pm^\dagger$. These states carry electric currents (loop currents). Performing the Peierls substitution, $t \to te^{ie\int \boldsymbol{A}\cdot d\boldsymbol{l}}$, and expanding at small B we find the magnetic moment per triangle

$$\mu_\pm = \pm\frac{etL^2}{16} \approx \pm 10\mu_B \tag{17}$$

The value corresponds to $L = 100$ nm and $t = 0.59$ meV.

To address the stability of the loop current state one needs to consider virtual hopping of an electron from one triangle to another. Here we follow the same approach as Anderson's superexchange for a Mott insulator. Hopping from one triangle to another creates two electrons on a triangle. The two-electron states are

$$\begin{aligned}
&\textit{parallel spins} \\
&|1\rangle = b_{+\uparrow}^\dagger b_{-\uparrow}^\dagger|0\rangle, \qquad E_1 = -2t + V_1 \\
&|2\rangle = b_{+\uparrow}^\dagger b_{0\uparrow}^\dagger|0\rangle, \qquad E_2 = t + V_1 \\
&\textit{antiparallel spins} \\
&|3\rangle = b_{+\uparrow}^\dagger b_{+\downarrow}^\dagger|0\rangle, \qquad E_3 = -2t + 2V_1/3 + U/3 \\
&|4\rangle = b_{+\uparrow}^\dagger b_{-\downarrow}^\dagger|0\rangle, \qquad E_4 = -2t + 2V_1/3 + U/3 \\
&|5\rangle = b_{+\uparrow}^\dagger b_{0\downarrow}^\dagger|0\rangle, \qquad E_5 = t + 2V_1/3 + U/3
\end{aligned} \tag{18}$$

Let us consider the loop current state $b_{+\beta}^\dagger a_{+\alpha}^\dagger$, where α and β are spin indexes. The energy of the state is

$$E_0 = -2t + V_2 \tag{19}$$

The hopping Hamiltonian $tb_{1\sigma}^\dagger a_{1\sigma}$ leads to mixing of the zero approximation wave function $b_+^\dagger a_+^\dagger$ with $b_+^\dagger b_\alpha^\dagger$, where $\alpha = +, -, 0$. There is also an equal contribution from $b \to a$ hopping. All in all

this leads to the second order perturbation theory energy shift δE (Anderson mechanism).

$$\begin{aligned} &\textit{parallel spins},\ \alpha=\beta: \qquad \delta E=-2(t/3)^2\left(\frac{1}{V_1-V_2}+\frac{1}{V_1-V_2+3t}\right) \\ &\textit{antiparallel spins},\ \alpha\neq\beta: \quad \delta E=-2(t/3)^2\left(\frac{2}{2V_1/3+U/3-V_2}+\frac{1}{2V_1/3+U/3+3t-V_2}\right) \end{aligned} \tag{20}$$

There are three conclusions from these equations

(i) The most "dangerous" term is that with the denominator $V_1 - V_2$, hence the stability of the state depends on the long-range interactions, the on-site Hubbard U is of secondary importance. For stability of the state one needs $(t/3)/(V_1 - V_2) \ll 1$, according to our estimates the value of the parameter is $(t/3)/(V_1 - V_2) \approx 0.25$.

(ii) The spin parallel orientation has lower energy than the antiparallel one. The energy difference is about 0.03 meV. Hence the spin dynamics are described by ferromagnetic spin Hamiltonian, $s_i = 1/2$,

$$\begin{aligned} &H_s = -J_s s_a s_b \\ &J_s \sim 0.06 \text{ meV} \sim 0.5 \text{ K} \end{aligned} \tag{21}$$

(iii) The energies (20) are independent of the relative orientation of loop currents, hence while at $T = 0$ spins are ordered ferromagnetically, the Ising orbital magnetic moments $\pm 10\mu_B$ remain fully disordered.

The energy splitting between different directions of the loop currents arises only with account of the next nearest hopping t'. The second order correction to the energy dependent on the relative current orientation arises due to the following sequences of hoppings, $3a \to 1b$ and then back $2b \to 1a$, also $1a \to 2b$ and then back $1b \to 3a$ (Fig. S20). For simplicity consider only ferromagnetic orientation of spins and account only for the virtual hoppings that have the minimal energy denominator, $\Delta E = V_1 - V_2$. This gives the following energy shifts: for the same direction of loop currents $\delta E = \frac{2(t'/3)^2}{V_1 - V_2}$, for opposite directions of loop currents $\delta E = -\frac{4(t'/3)^2}{V_1 - V_2}$. Hence the antiferromagnetic ordering of the loop currents is preferable and the effective Hamiltonian of Ising moments $l_i = \pm 1$ is

$$\begin{aligned} &H_l = J_l l_a l_b \\ &J_l \approx \frac{1}{3}\frac{(t')^2}{V_1 - V_2} \sim 0.002 \text{ meV} \sim 20 \text{ mK} \end{aligned} \tag{22}$$

Since J_l is very small the loop currents are practically always disordered at $B = 0$. However, an application of an external out of plane magnetic field orders the currents since each loop carries

magnetic moment $\sim 10\mu_B$. Electrons thermally excited over the gap to the "conduction band" provide conductivity. They scatter from thermal fluctuations of the loop currents, ordering of the currents by magnetic field reduces the fluctuations in this results in the giant negative magnetoresistance.

---

[1] D. Weiss, P. Grambow, K. von Klitzing, A. Menschig, and G. Weimann, Applied Physics Letters **58**, 2960 (1991).

[2] G. De Simoni, A. Singha, M. Gibertini, B. Karmakar, M. Polini, V. Piazza, L. N. Pfeiffer, K. W. West, F. Beltram, and V. Pellegrini, Applied Physics Letters **97**, 132113 (2010).

[3] A. Sandner, T. Preis, C. Schell, P. Giudici, K. Watanabe, T. Taniguchi, D. Weiss, and J. Eroms, Nano Letters **15**, 8402 (2015).

[4] R. Yagi, R. Sakakibara, R. Ebisuoka, J. Onishi, K. Watanabe, T. Taniguchi, and Y. Iye, Phys. Rev. B **92**, 195406 (2015).

[5] E. C. Peters, A. J. M. Giesbers, U. Zeitler, M. Burghard, and K. Kern, Phys. Rev. B **87**, 201403 (2013).

[6] J. Pan, T. Zhang, H. Zhang, B. Zhang, Z. Dong, and P. Sheng, Phys. Rev. X **7**, 031043 (2017).

[7] B. S. Jessen, L. Gammelgaard, M. R. Thomsen, D. M. A. Mackenzie, J. D. Thomsen, J. Caridad, E. Duegaard, K. Watanabe, T. Taniguchi, T. J. Booth, T. G. Pedersen, A.-P. Jauho, and P. Bøggild, Nature Nanotechnology **14**, 340 (2019).

[8] C. Forsythe, X. Zhou, K. Watanabe, T. Taniguchi, A. Pasupathy, P. Moon, M. Koshino, P. Kim, and C. R. Dean, Nature Nanotechnology **13**, 566 (2018).

[9] R. Huber, M. N. Steffen, M. Drienovsky, A. Sandner, K. Watanabe, T. Taniguchi, D. Pfannkuche, D. Weiss, and J. Eroms, Nature Communications **13**, 2856 (2022).

[10] M. Yankowitz, J. Xue, D. Cormode, J. D. Sanchez-Yamagishi, K. Watanabe, T. Taniguchi, P. Jarillo-Herrero, P. Jacquod, and B. J. LeRoy, Nature Physics **8**, 382 (2012).

[11] Y. Cao, V. Fatemi, A. Demir, S. Fang, S. L. Tomarken, J. Y. Luo, J. D. Sanchez-Yamagishi, K. Watanabe, T. Taniguchi, E. Kaxiras, R. C. Ashoori, and P. Jarillo-Herrero, Nature **556**, 80 (2018).

[12] S. Wang, D. Scarabelli, L. Du, Y. Y. Kuznetsova, L. N. Pfeiffer, K. W. West, G. C. Gardner, M. J. Manfra, V. Pellegrini, S. J. Wind, and A. Pinczuk, Nature Nanotechnology **13**, 29 (2018).

[13] L. Du, Z. Liu, S. Wind, V. Pellegrini, K. West, S. Fallahi, L. Pfeiffer, M. Manfra, and A. Pinczuk, Physical Review Letters **126**, 106402 (2021).

[14] O. A. Tkachenko, V. A. Tkachenko, I. S. Terekhov, and O. P. Sushkov, 2D Materials **2**, 014010 (2015).

[15] J. P. Bird, A. D. C. Grassie, M. Lakrimi, K. M. Hutchings, J. J. Harris, and C. T. Foxon, Journal of Physics: Condensed Matter **2**, 7847 (1990).

[16] P. A. Lee, A. D. Stone, and H. Fukuyama, Phys. Rev. B **35**, 1039 (1987).

[17] C. M. Varma, Phys. Rev. B **55**, 14554 (1997).

[18] C. M. Varma, Rev. Mod. Phys. **92**, 031001 (2020).
